# Shorebirds’ Longer Migratory Distances Are Associated With Larger *ADCYAP1* Microsatellites and Greater Morphological Complexity of Hippocampal Astrocytes

**DOI:** 10.3389/fpsyg.2021.784372

**Published:** 2022-02-04

**Authors:** Diego de Almeida Miranda, Juliana Araripe, Nara G. de Morais Magalhães, Lucas Silva de Siqueira, Cintya Castro de Abreu, Patrick Douglas Corrêa Pereira, Ediely Pereira Henrique, Pedro Arthur Campos da Silva Chira, Mauro A. D. de Melo, Péricles Sena do Rêgo, Daniel Guerreiro Diniz, David Francis Sherry, Cristovam W. P. Diniz, Cristovam Guerreiro-Diniz

**Affiliations:** ^1^Instituto Federal de Educação Ciência e Tecnologia do Pará, Campus Bragança, Laboratório de Biologia Molecular e Neuroecologia, Bragança, Brazil; ^2^Laboratório de Genética e Conservação, Instituto de Estudos Costeiros (IECOS), Universidade Federal do Pará, Bragança, Brazil; ^3^Laboratório de Investigações em Neurodegeneração e Infecção, Instituto de Ciências Biológicas, Universidade Federal do Pará, Hospital Universitário João de Barros Barreto, Belém, Brazil; ^4^Laboratório de Microscopia Eletrônica, Instituto Evandro Chagas, Belém, Brazil; ^5^Department of Psychology, Advanced Facility for Avian Research, University of Western Ontario, London, ON, Canada

**Keywords:** migratory birds, *ADCYAP1* microsatellites, GFAP astrocytes morphological complexity, migratory distance, migration

## Abstract

For the epic journey of autumn migration, long-distance migratory birds use innate and learned information and follow strict schedules imposed by genetic and epigenetic mechanisms, the details of which remain largely unknown. In addition, bird migration requires integrated action of different multisensory systems for learning and memory, and the hippocampus appears to be the integration center for this task. In previous studies we found that contrasting long-distance migratory flights differentially affected the morphological complexity of two types of hippocampus astrocytes. Recently, a significant association was found between the latitude of the reproductive site and the size of the *ADCYAP1* allele in long distance migratory birds. We tested for correlations between astrocyte morphological complexity, migratory distances, and size of the *ADCYAP1* allele in three long-distance migrant species of shorebird and one non-migrant. Significant differences among species were found in the number and morphological complexity of the astrocytes, as well as in the size of the microsatellites of the *ADCYAP1* gene. We found significant associations between the size of the *ADCYAP1* microsatellites, the migratory distances, and the degree of morphological complexity of the astrocytes. We suggest that associations between astrocyte number and morphological complexity, *ADCYAP1* microsatellite size, and migratory behavior may be part of the adaptive response to the migratory process of shorebirds.

## Introduction

Overall, long-distance navigation includes at least three different phases. The first involves long distance guidance based on global tracks (Able, 1991; Wiltschko R. and Wiltschko, 2012; Wiltschko W. and Wiltschko, 2012; Wiltschko and Wiltschko, 2019); the second includes the construction of a variety of local gradient maps based on learned information from all available sensory information (Bingman and MacDougall-Shackleton, 2017). The last involves identifying the target area, most likely based on local landmarks (Frost and Mouritsen, 2006; Bingman and MacDougall-Shackleton, 2017). In young birds on their first migratory journey, navigation is based on compass-*CLOCK* orientation that requires only an inherited migratory direction, a circannual *CLOCK* and at least one compass (van Toor et al., 2013; Mouritsen et al., 2016). In experienced birds, in addition to the compass-*CLOCK* system, learned maps become part of the navigation system (Mouritsen et al., 2016) and include olfactory cues (Gagliardo et al., 2013), landmarks (Mann et al., 2014), celestial tracks (Emlen, 1975), and geomagnetic signals (Dennis et al., 2007).

Specific genes or gene regions, and polymorphisms at candidate genes, may explain phenotypic variance in many aspects of migratory behavior (Bazzi et al., 2016b). Microsatellite loci show high mutation rates with a high level of polymorphism with significant changes in the number of repeats and allele size (Hardy et al., 2003) and have been widely used for investigation of genetic patterns among and within populations (Balloux and Lugon-Moulin, 2002; Song et al., 2011). Indeed, after investigate polymorphisms in the exon of six candidate genes for a relationship with behavioral migratory phenotypes a consistent association there was found a consistent association between microsatellite polymorphism at only one candidate gene: the *ADCYAP1* (Mueller et al., 2011). Other studies on neotropical migratory passerines (*Passerina ciris*) however, have pointed out that the size of microsatellite alleles of the *ADCYAP1* and *CLOCK* gene do not show correlation with the onset or duration of migration, making this a controversial issue (Contina et al., 2018). In contrast, in blackpoll warblers (*Setophaga striata*), *Clock* and *ADCYAP1* allele lengths were correlated with migratory behaviors (Ralston et al., 2019). In addition, it has been suggested that potential interaction between *ADCYAP1*, wing morphology and sex predict spring migration arrival in blackcap (*Sylvia atricapilla*) populations (Mettler et al., 2015) and that both *ADCYAP1* and *CLOCK* gene alleles increase in size with breeding latitude in trans-Saharan migratory birds (Bazzi et al., 2016a,b).

Thus, it emerges that we are still far from filling in the details of the functional contribution of the *ADCYAP1* and *CLOCK* genes to the intricate interaction between the internal clock and environmental conditions regulating the annual navigation cycle of long-distance migratory birds (Åkesson et al., 2017).

The adenylate cyclase activating polypeptide 1 (*ADCYAP1*) is a dinucleotide microsatellite locus in the 3′ UTR (untranslated region) of avian chromosome 2a with polymorphism associated with phenotypic variance of migratory behavior (Bazzi et al., 2016b; Contina et al., 2018). It is a protein code gene with polymorphic profile, which encodes the pituitary adenylate cyclase activator peptide (PACAP), widely expressed in the central nervous system (CNS) and peripheral organs (Montero et al., 2000; Gahete et al., 2009; Toth et al., 2020). PACAP alters neurotransmitter release and contributes to regulation of energy homeostasis in the CNS acting within hypothalamic/hypophyseal system (Rudecki and Gray, 2016; Gastelum et al., 2021), and through limbic actions that contribute to cognition (Kirry et al., 2018; Ciranna and Costa, 2019; Gilmartin and Ferrara, 2021). In the periphery it causes increased insulin (Shao et al., 2013) and histamine (Schubert, 2003) secretions, controls vasodilation (Baliga et al., 2013), bronchodilation (Lindén et al., 1999; Kinhult et al., 2000), modulates innate and adaptive immunity (Ganea and Delgado, 2002), alters intestinal motility (Bornstein et al., 2004) and stimulates cellular proliferation as well as differentiation (Nowak and Zawilska, 2003; Vaudry et al., 2009; Prisco et al., 2019; Karpiesiuk and Palus, 2021). In the CNS, PACAP displays pleiotropic activity, including functions as a hypophysiotropic hormone (Vélez and Unniappan, 2020), neuromodulator, and neurotrophic factor (Sadanandan et al., 2021). PACAP is also involved in the rhythmicity of melatonin production and in the increase of cAMP in birds (Nakahara et al., 2002).

*ADCYAP1* gene encodes pituitary adenylate cyclase-activating polypeptide (Bakalar et al., 2021) which contributes to energy homeostasis (Rudecki and Gray, 2016; Bozadjieva-Kramer et al., 2021; Gastelum et al., 2021), and astroglial functions are modulated by PACAP (Masmoudi-Kouki et al., 2007; Hansson et al., 2009; Nakamachi et al., 2011; Kong et al., 2016; Seo and Lee, 2016; Kambe et al., 2021), and respond to many metabolic demands regulating a wide array of physiological processes (Murat and García-Cáceres, 2021) including hippocampal-dependent behavioral functions (Johnson et al., 2020).

We previously explored how the contrasting navigation strategies of the semipalmated sandpiper (*Calidris pusilla*) and the semipalmated plover (*Charadrius semipalmatus*) during autumn migration were related to hippocampal astroglia morphology (Carvalho-Paulo et al., 2018; Mendes de Lima et al., 2019; Henrique et al., 2020). This comparative analysis of morphological features to classify astrocytes revealed there were two types of morphological astrocytes influenced in different ways by contrasting long-distance migratory flights suggesting distinct physiological roles for these cells (Carvalho-Paulo et al., 2018; Henrique et al., 2020).

Because the hippocampus integrates all information related to bird’s migratory behavior (Mouritsen et al., 2016; Bingman and MacDougall-Shackleton, 2017) we searched in this exploratory investigation for an association between astrocyte morphological complexity, size of microsatellites of the *ADCYAP1* allele and migratory distances in four species: the spotted sandpiper (*Actitis macularius*), the semipalmated sandpiper (*C. pusilla*), the semipalmated plover (*C. semipalmatus*), and the non-migratory collared plover (*Charadrius collaris*).

We hypothesized that astrocyte morphological complexity, migratory distances, and size of the *ADCYAP1* allele will be correlated and to test this hypothesis we selected three long-distance migrant species of shorebird and one non-migrant.

## Materials and Methods

### Sampling Area

For both *ADCYAP1* analysis and astrocyte morphometry, we collected adult individuals with mist-nets during the wintering period in the mangroves of the Amazon River estuary. All individuals were collected between 2012 and 2017, in the northeast of Pará state, at the municipality of Bragança, Pará, Brazil, on Canela Isle (0° 47′33.52″ S 46° 43′ 8.55″ W), Lombo Grande Isle (0 ° 47′33.52″ S 46 ° 43′8.55″ W), Praia do Pilão (0 ° 47′46.08″ S 46 ° 40′29.64″ W), Baiacu Beach (0 ° 47′32.55″ S 46 ° 46′52.05″ W), Quatipuru Mirim Beach (0 ° 46′35.61″ S 46 ° 52′57.66″ W) and Otelina Isle (0 ° 45′42.57″ S 46 ° 55′ 51.86″ W). Captured wintering birds included *C. semipalmatus*, *C. pusilla*, and *A. macularius*. The non-migratory *C. collaris*, a resident of South America, was also captured and compared with the long-distance migratory species (Del Hoyo et al., 1992; Rodrigues, 2000, 2006). Immediately after capture, biometric data were obtained from all individuals.

Birds were captured in compliance with license No. 44551-2 of the Chico Mendes Institute for Biodiversity Conservation (ICMBio), minimizing discomfort during handling as much as possible.

*Actitis macularius* presents a pattern of migration on broad fronts with many stopover sites and a broad dispersion on spring and summer grounds, whereas *C. pusilla* has a narrow band of migration and moderate dispersion on spring and summer sites (Reed and Oring, 1993; Billerman et al., 2020). Migration timing for these species is very similar but greater migratory distance is performed by *C. pusilla* compared to *A. macularius* (Skagen et al., 1999). *C. semipalmatus* flies towards coastal areas in the southern United States, the Caribbean and much of South America. Like *A. macularius*, *C. semipalmatus* travels long distances with flights interrupted for resting and feeding (Campos et al., 2008; Serrano, 2010; Nóbrega et al., 2015).

### *ADCYAP1*: Microsatellite Genotyping

Microsatellites have been used in ecological and conservation studies since the 1990s (Moura et al., 2017). The comparison between the length of the alleles and the nucleotide composition of the base of the sequence makes it possible to identify variation in the microsatellites (Ellegren, 2004). Allele size variation is identified by polymerase chain reaction (PCR) amplification (Primmer et al., 1996). In the present report we carried out DNA isolation and purification from stored blood samples of 53 individuals with distinct migratory behaviors: *A. macularius* (*n* = 12), *C. pusilla* (*n* = 14), *C. semipalmatus* (*n* = 13), and *C. collaris* (*n* = 14). After blood collection (less than 100 μL), all captured animals were released back into the wild, except for five individuals of each species used in morphometric studies. We followed the recommendations of the DNA extraction protocol of Wizard^®^ Genomic Purification Kit (PROMEGA).

For PCR, specific primers were used that flank microsatellite repetitions of the *ADCYAP1* locus (Steinmeyer et al., 2009). To amplify the *ADCYAP1* loci, the M13 tail technique proposed by Schuelke (2000) was used. The principle of the technique is to use primer pairs that flank repetitive DNA sequences to amplify samples of genomic DNA and to examine the size of the amplified alleles on a sequencing device (Boutin et al., 1997; Boutin-Ganache et al., 2001). The PCR reaction was performed using a total volume of 13 μL containing 5 ng of DNA, 10 μL Buffer, 1.5 mM MgCl_2_, 1.2 mM dNTP, 8 pM M13 probe and reverse primer, 0.5 pM of primer forward and 1 unit (U) of Taq DNA polymerase. To find the best hybridization temperature for the studied species, PCRs were performed with temperature gradients between 50 and 60°C. The PCR reaction consisted of an initial denaturation of 94°C for 5min, followed by 30 cycles of 94°C for 30s, 51°C for 45s and 72°C for 45s, followed later by 8 cycles of 94°C for 30s, 53°C for 45s and 72°C for 45s, with a final extension of 72°C for 10min [for more details on PCR see Schuelke (2000)]. We used the standard microsatellite genotyping method in the ABI 3500XL fragment analyzer (GeneMapper, Applied Biosystems). Subsequently, peak patterns were analyzed by the Fragment Profiler 1.2 program (Amersham Biosciences) and organized in Microsoft Excel 2019.

To detect and identify genotyping errors resulting from null alleles, allele drop out and stuttering related to *ADCYAP1* locus, we used the software MICROCHECKER (Barros et al., 2020). We used ARLEQUIN v3.5 (Excoffier et al., 2007) to measure genetic diversity in terms of number of alleles per locus (A), observed (HO), and expected (HE) heterozygosity (Nei, 1978). We tested for Hardy–Weinberg Equilibrium (Mayo, 2008; Meirmans et al., 2018) and analyzed population differentiation at gene *ADCYAP1* through FST and RST statistics (Meirmans, 2020) with a significance level α < 0.05.

### Immunohistochemistry

Five individuals of each species were used for morphometric astrocyte studies. All birds were anesthetized with Isoflurane (Olkowski and Classen, 1998), euthanized with an anesthetic overdose, and perfused transcardially with 0.1% heparinized phosphate buffered saline (PBS) for 10 min, followed by 4% paraformaldehyde pH 7.2–7.4 for another 30 min. After craniotomy, brains were removed and stored in 9% phosphate buffer (Sigma Aldrich - S3264) and then cut in the coronal plane. Eighty micrometer thick sections were obtained using a vibrating blade microtome (LeicaVibratomeVT1000S). Serial anatomical sections (1:6 interval) were subjected to immunohistochemical reactions using GFAP selective marker for astrocytes.

### Three-Dimensional Reconstruction

In previous reports we used three-dimensional microscopic reconstructions and applied hierarchical cluster analysis to classify astrocytes based on morphometric features using the largest Euclidean distance between the groups. We found two large morphological families (Type I and Type II). These families were differentially affected by contrasting migratory patterns (Carvalho-Paulo et al., 2018; Henrique et al., 2020). Here, we performed hierarchical clustering on variance-shrunk logarithmized values of multimodal morphometrical features to define the number of morphological families and compared hippocampal astrocyte morphologies of long-distance migratory birds with contrasting migratory flights (*C. semipalmatus*, *C. pusilla*, and *A. macularius*) with a non-migratory specie (*C. collaris*).

For the three-dimensional reconstruction of positive GFAP astrocytes, an optical microscope (Eclipse Ci, NIKON) with motorized stage and analog-digital converters (MAC6000 System, Ludl Electronic Products, Hawthorne, NY, United States) was used. This system was coupled to a microprocessor that controlled the movements of the microscopic stage with the aid of a specialized program (Neurolucida, MBF Bioscience, Williston, VT, United States) to store the spatial information (X, Y, Z coordinates) of each digitized point of interest. Three types of cells were identified: protoplasmic, fibrous, and radial astrocytes similar to previous descriptions (Carvalho-Paulo et al., 2017; Mendes de Lima et al., 2019; Henrique et al., 2020). In this study, we used only protoplasmic astrocytes.

The contours of the hippocampal formation (Atoji and Wild, 2004; Atoji et al., 2016) were determined on a low power objective (4× lens). To identify astrocyte morphological details and to ensure greater detail in 3D reconstructions, the low power lens was replaced by a high-power oil immersion 100× lens PLANFLUOR (NA 1.3; DF = 0.2 μm; Nikon, Japan).

**FIGURE 1:**
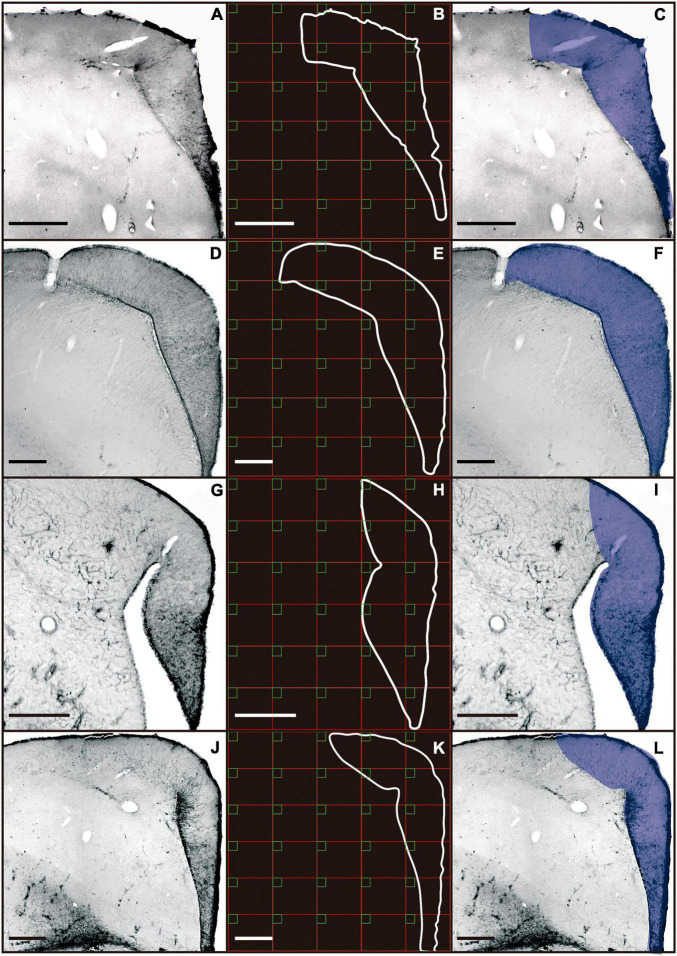
Photomicrographs of coronal sections of the telencephalon of *C. collaris* **(A)**, *C. semipalmatus* **(D)**, *C. pusilla* **(G)**, and *A. macularius* **(J)**. Outline of the hippocampal formation, and sampling grid **(B,E,H,K)**. Borders of the hippocampal formation are defined by blue line **(C,F,I,L)**. Scale bars: **(A)** 250 μm; **(D)** 500 μm; **(G)** 500 μm; **(J)** 500 μm.

A total of 264 astrocytes from the hippocampal formation of *A. macularius*, 251 of *C. pusilla*, 302 of *C. semipalmatus*, and 260 of *C. collaris* were reconstructed in three dimensions. To select astrocytes for reconstruction a random and systematic stereological sampling approach was adopted (West, 2002). For this, we used squared probes (50 μm × 50 μm) separated from each other by a 900 μm × 900 μm grid interval (Figure 1). The grid interval was estimated to obtain a minimum of 50 reconstructed astrocytes per animal and it was selected based on the total area of the hippocampal formation. The number of probes per section was proportional to the area of the hippocampal formation of each section.

Only astrocytes located within the limits of the probe box were selected for analysis. In cases where there were no cells within the probe box that met the pattern of complete immunostaining and branch integrity, the closest astrocyte outside the probe box border was chosen for reconstruction. Due to minimal shrinkage in the X/Y axes, the correction for shrinkage induced by histological processing was applied exclusively for the z axis, and corresponded to 1.75×, as previously recommended by Carlo and Stevens (2011).

Supplementary Table 1 shows the definition of all morphometric features of hippocampal astrocytes. The following equation adapted from previous neuronal dendritic reconstruction (Pillai et al., 2012) was used to estimate morphological complexity:

Complexity =[Sum of the terminal orders + Number of terminals] × [Total branch length/Number of primary branches]

Thus, 20 morphological variables were used for morphometry: total branch length, branch surface area, tortuosity, total branch volume, base diameter of primary branches, total number of segments, segments/mm, number of branching points, tree surface area (μm^2^), planar angle, complexity, convex hull perimeter (μm), convex hull area (μm^2^) 2-D, convex hull surface area (μm^2^), convex hull volume (μm^3^), and Vertex: Va, Vb, Vc, K-dim (fractal dimension) (see Supplementary Table 1 for details).

### Statistical Analysis

#### Morphometry

With a total of 1,077 reconstructed astrocytes from groups of migrant and non-migrant birds, we performed a multivariate statistical analysis using 20 morphological parameters. This procedure was used to identify possible morphological clusters within each species (Schweitzer and Renehan, 1997; Yamada and Jinno, 2013). Morphometric data for all astrocytes were obtained using Neuroexplorer software (MicroBright Field Inc.). To search for morphological characteristics shared by astrocytes, only quantitative morphometric variables with multimodality indices (MMI) greater than 0.55 were selected, to identify which variables were multimodal or at least bimodal. MMI was estimated based on the parameters of asymmetry and kurtosis of each morphometric variable, *MMI* = [*M*3^2^+1]/[*M*4+3(*n*−1)^2^/(*n*−2)*n*−3)] in which M3 is asymmetry, M4 is kurtosis and *n* is the sample size (Schweitzer and Renehan, 1997).

Hierarchical cluster analysis using Ward’s method or Ward’s Minimum Variance Clustering Method (Ward, 1963) was applied to multimodal variables to classify cells (Schweitzer and Renehan, 1997). Variance-shrunk logarithmized values of multimodal morphometrical features were then submitted to cluster analysis. The morphometric variables used in the cluster analysis (MMI > 0.55) were subjected to discriminant analysis, using Statistica 12.0 software. This procedure identifies which variables contribute most to the formation of clusters. The software compares matrices of total variances and covariances using multivariate F tests to determine if there are significant differences between groups (for all variables). In the analysis of the step-forward discriminant function, the program builds a step-by-step discrimination model. In this model, at each test stage, all variables are reviewed and evaluated to determine which variable contributes the most to discrimination between groups. If any variable did not have a *p*-value below 0.05 or did not occur in all studied species, it was disregarded in the subsequent analyses so that we could detect the morphometric variables that provided the best separation between the astrocyte morphological classes suggested by the cluster analysis.

Following discriminant analysis, we found morphological complexity and convex hull volume to be the variables shared by all species that contributed most to cluster formation. We did an initial test of normality (Shapiro–Wilk) (Shapiro and Wilk, 1965) and homogeneity of variances (Levene, 1960) (Supplementary Table 2) and then transformed the scale of complexity values into decimal log units to perform an independent univariate General Linear Model (GLM) test (Nelder and Wedderburn, 1972). We used the Sidak test (Salkind, 2007) for paired tests, and for effect size used the Cohen d test (Thalheimer and Cook, 2002).

For a priori multivariate comparison tests, data with continuous variables were transformed into values of Log (X + 1) (Clarke and Warwick, 2001) and normalized (Anderson et al., 2008) to correct additivity effects of factor and scale diversity, respectively. Then we generated Euclidean similarity matrix where the data were exchanged to verify significant differences (setting α = 0.05) through dispersion homogeneity tests (PERMIDISP) (Anderson et al., 2006) and the Analysis of Variance by Multivariate Permutation (PERMANOVA) (Anderson et al., 2008).

In PERMIDISP, we verified whether the differences found were associated with sample dispersion using the distance protocol between the centroids with 9,999 permutations and paired tests. In order to have the values of pseudo-F in PERMANOVA, we verified possible differences in the location of the samples by treating the distance matrices using the residual permutation method under a reduced model (“residuals under a reduced model”) with 9,999 repetitions, sum of squares “type III” (McArdle and Anderson, 2001) and paired tests (pseudo-t). The tests were considered two factor (“species” and “type”) and treated as fixed. All tests were generated using the PRIMER E software (Anderson et al., 2008).

#### Analysis of *ADCYAP1* Microsatellites

**FIGURE 2:**
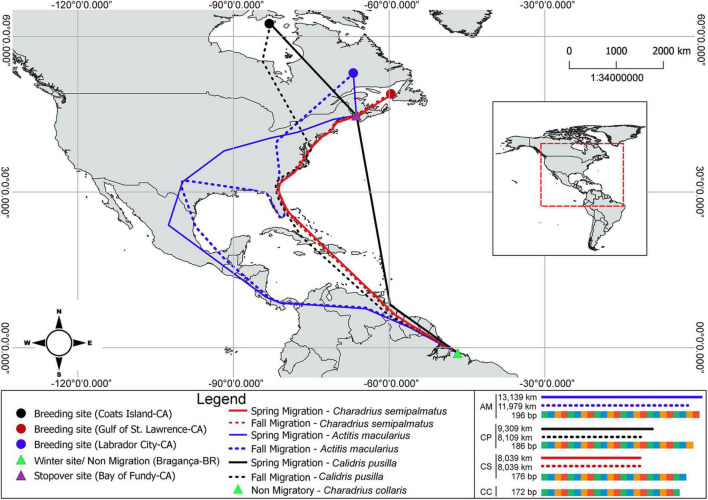
Breeding sites and migratory routes to the wintering area of *C. semipalmatus*, *C. pusilla*, and *A. macularius.* Lower right panel shows the size of the *ADCYAP1* gene.

The migration distance traveled by each migrant species was estimated using information available on the departure of birds from North America (breeding site) to their arrival at the isles of Bragança estuarine region in South America (wintering site) (Reed et al., 2013; Nóbrega et al., 2015; Billerman et al., 2020; Hicklin and Gratto-Trevor, 2020). *C. semipalmatus*, *C. pusilla*, and *A. macularius* are long-distance migratory birds whereas *C. collaris* is classified as a resident species of South America (Piersma and Wiersma, 1996). *C. semipalmatus* travels around 8,039 km, while *C. pusilla* travels 9,309 km and *A. macularius* around 13,139 km. We adopted 0 (zero) for the migratory route of *C. collaris* (Reed et al., 2013; Nóbrega et al., 2015; Billerman et al., 2020; Hicklin and Gratto-Trevor, 2020) (see Figure 2).

Because our samples were small (*n* < 30) and the data did not follow a normal distribution, a Spearman rank correlation coefficient was calculated to assess the degree of correlation between migratory distance and size of the microsatellites.

To detect differences in the size of the microsatellites between the species, PERMANOVA was performed, as an analogue to univariate ANOVA. We followed the same criteria described above in the morphometric analyses. For this test, however, we used the residual exchange method under an unrestricted model with 9,999 repetitions with sum of squares for “Type III” (McArdle and Anderson, 2001) in the PRIMER E software (Anderson et al., 2008).

### Photomicrographs and Post-Processing

For photomicrographs, a digital camera (Microfire, Optronics, CA, United States) coupled with a Nikon microscope (Eclipse Ci, NIKON) was used, and acquired images were post-processed for brightness and contrast with Adobe Photoshop software (Adobe Inc San José, CA, United States). We selected images of the most representative astrocytes of each cell type indicated by the hierarchical cluster analysis. For the choice of the representative cell of each group (“average cell”), the distance matrix was used to obtain the sum of the distances of each cell relative to all others. It is assumed that the cell that best represents a group has the smallest sum of distances. The matrices were constructed with the combination of all cells of a given group taken pairwise, followed by the weighted calculation of a scalar Euclidean distance between cells using all morphometric variables (Henrique et al., 2020).

## Results

### Size of Microsatellites in ADCYPA1

We identified 17 alleles for the *ADCYAP1* locus, varying in size from 168 to 204 base pairs (bp). The most common alleles found were 172 and 174 bp. The *ADCYAP1* locus was polymorphic in all four species with the number of alleles varying from 5 to 6, and number of exclusive alleles varying from 1 to 4 across populations (Supplementary Table 3). Analysis showed no evidence of null alleles and genotype errors such as stuttering, or allele drop out. However, two out of the four populations exhibited significant deviation (*p* < 0.01) from Hardy–Weinberg equilibrium and higher numbers of homozygotes compared to the other populations. Observed and expected heterozygosity ranged from 0.417 to 0.786 and from 0.712 to 0.792, respectively (Supplementary Table 4).

All pairwise F_st_ and R_st_ values based on *ADCYAP1* allele frequencies presented significant differentiation across species, which was expected since all are distinct and known species (Supplementary Table 5). Higher levels of genetic differentiation were observed across species pairwise comparisons and low levels were observed between species in the *Charadrius* genus, corroborating the evolutionary relations of these groups.

**FIGURE 3:**
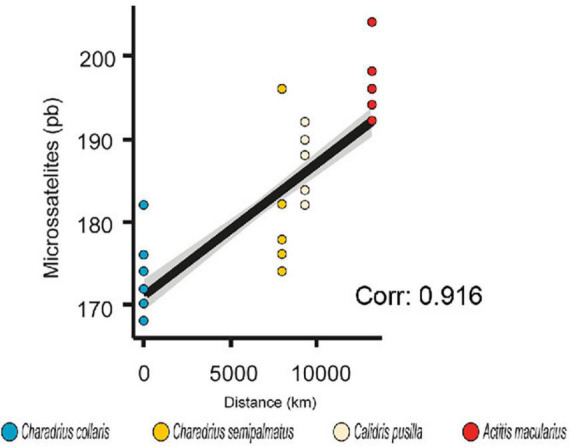
Comparison and correlation tests in microsatellites with migratory distance. Linear regression (R = 0.840) and Spearman’s correlation (Rho = 0.916) showing positive association between size of *ADCYAP1* alleles as pair of bases (pb) and migratory distance.

The association of size of microsatellites with migratory distance showed a positive and strongly supported association (Spearman correlation test, Rho = 0.915; *p* = 0.000; Figure 3 and Supplementary Table 6).

We also performed a comparison between the size of the microsatellites in the four species (Supplementary Table 7). We found significant differences (PERMANOVA, *F* = 180.98, *p* = 0.0001) in all possible comparisons (*C. semipalmatus* vs *C. collaris*, *p* < 0.026; *C. semipalmatus* vs *C. pusilla*, *p* < 0.025; *C. semipalmatus* vs *A. macularius*, *p* < 0.0001; *C. collaris* vs *C. pusilla*, *p* < 0.0001; *C. collaris* vs *C. pusilla*, *p* < 0.0001; *A. macularius* vs *C pusilla*, *p* < 0.0127).

### Three-Dimensional Reconstruction of Astrocytes in Hippocampal Formation

**FIGURE 4:**
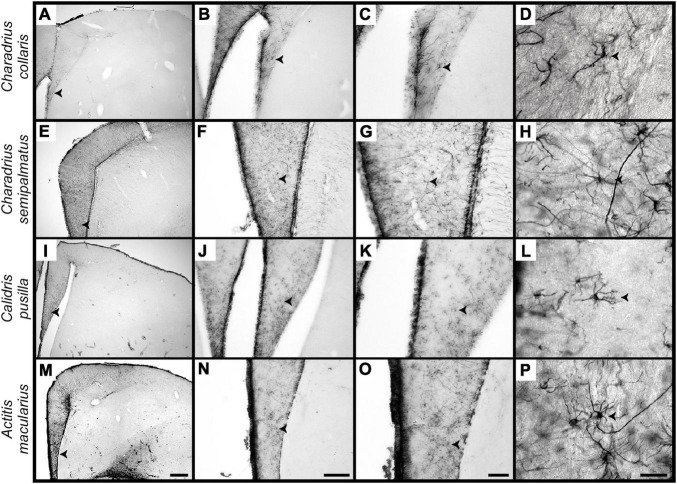
Photomicrographs of brain sections to illustrate stellate astrocytes of the hippocampus V region of non-migrant (*C. collaris*) and migrant (*C. semipalmatus, C. pusilla*, and *A. macularius*) species. From top to bottom rows correspond to *C. collaris, C. semipalmatus, C. pusilla*, and *A. macularius*. **(A–P)** Are photomicrographs from hippocampal sections of *C. collaris*, *C. semipalmatus, C. pusilla* and *A. macularius*, respectively, to illustrate GFAP stellate astrocytes of each species at different magnifications. Detailed three-dimensional reconstructions of these cells were done using high-power (100x) microscope lens. Scale bars: **(A,B,G,L)** = 250 mm; **(F,K,P)** = 500 mm; **(C,H,M)** = 120 mm and **(D,I,N)** = 60 mm; **(E,J,O)** = 25 mm.

Stellate astrocytes in the hippocampal formation of *A. macularius*, *C. pusilla*, *C. semipalmatus*, and *C. collaris* show glial fibrillary acidic protein expression in the cell body, from which GFAP positive primary thicker branches emerge and progressively ramify, terminating as tiny branches (Figure 4).

**FIGURE 5:**
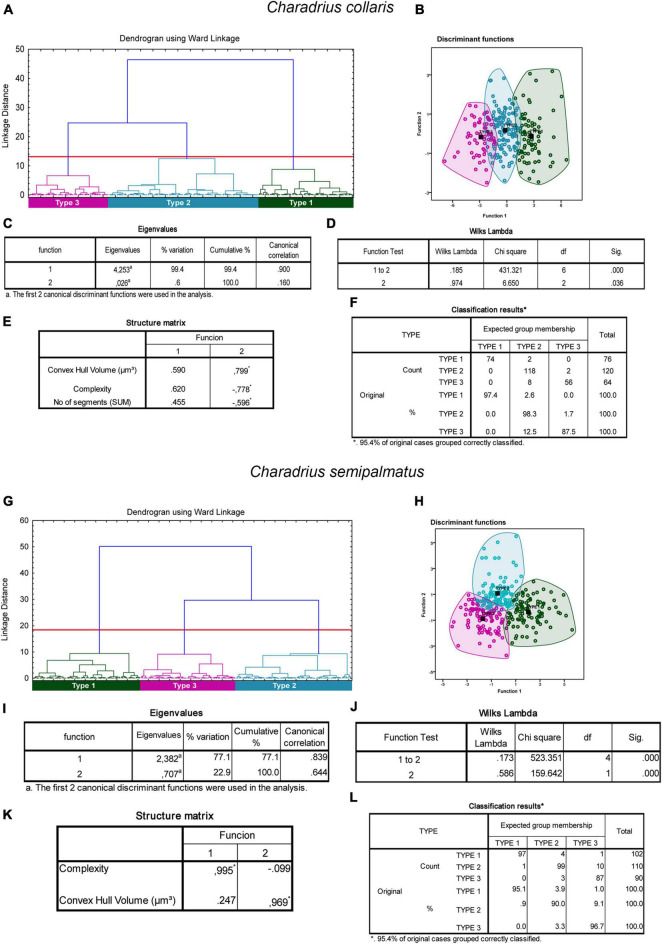
Cluster analysis of the morphology of astrocytes from rostral, intermediate, and caudal regions of the hippocampal formation of *Charadrius collaris* **(A–F)** and *Charadrius semipalmatus* **(G–L)**. Dendrogram representation of the hierarchical cluster analysis (Ward’s method) of the 260 cells of five individual *C. collaris* **(A)**. Notice three main morphological phenotypes named Type I (green), Type II (blue), and Type III (magenta). Graphic representation of the canonical discriminant function analysis illustrates the distribution of the three main clusters of astrocytes in the Euclidean space **(B)**. Colored circles green, blue, and magenta identify individual astrocytes of Type I, Type II, and Type III morphotypes. The variables that most contributed to the cluster’s formation were morphological complexity and convex hull volume. The eigenvalues output (C) indicates that function 1 explains 99.4% of the variance. Wilks’s lambda values in the function test 1–2, rejected the null hypothesis of no differences between the groups for these 2 variables (0.185; *p* = 0.000). In the structure matrix panel **(E)** is displayed the relative contributions of the variables to the cluster formation of functions 1 and 2. **(G)** Dendrogram representation of hierarchical cluster analysis (Ward’s method) of 302 reconstructed astrocytes of *C. semipalmatus* (*n* = 5). Three main morphological phenotypes named Type I (green), Type II (blue), and Type III (magenta) were found. Graphic representation of the canonical discriminant function analysis illustrates the distribution of the three main clusters of astrocytes in the Euclidean space **(H)**. Colored circles green, blue, and magenta identify individual astrocytes of Type I, Type II, and Type III morphotypes. The variables that most contributed to the cluster’s formation were morphological complexity and convex hull volume. The eigenvalues output (I) indicates that function 1 explains 77.1% of the variance. Wilks’s lambda values in the function test 1–2 **(J)**, rejected the null hypothesis of no differences between the groups for these 2 variables (0.173; *p* = 0.000). In the structure matrix panel (K) is displayed the relative contributions of the variables to the cluster formation of functions 1 and 2. Classification results for the expected group membership **(F)**. Black squared dots indicate de centroid of each cluster. (*) indicates statistically significant difference.

From hierarchical cluster and discriminant function analysis emerged that in the non-migratory species, the morphological complexity, the volume of the convex hull and the number of segments were the multimodal variables that most contributed to the formation of clusters (Figures 5A,B). In the analysis of the canonical discriminant function, it was shown that the complexity and volume of the convex hull accounted for 99.4% of the variance (Figures 5C,D). Wilks’s lambda value correspondent to 1 and 2 canonical discriminant functions was the most important for group separation (Wilks’s lambda = 0.185; *p* = 0.000) (Figure 5E) with 95.4% of the group data classified correctly (Figure 5F).

**FIGURE 6:**
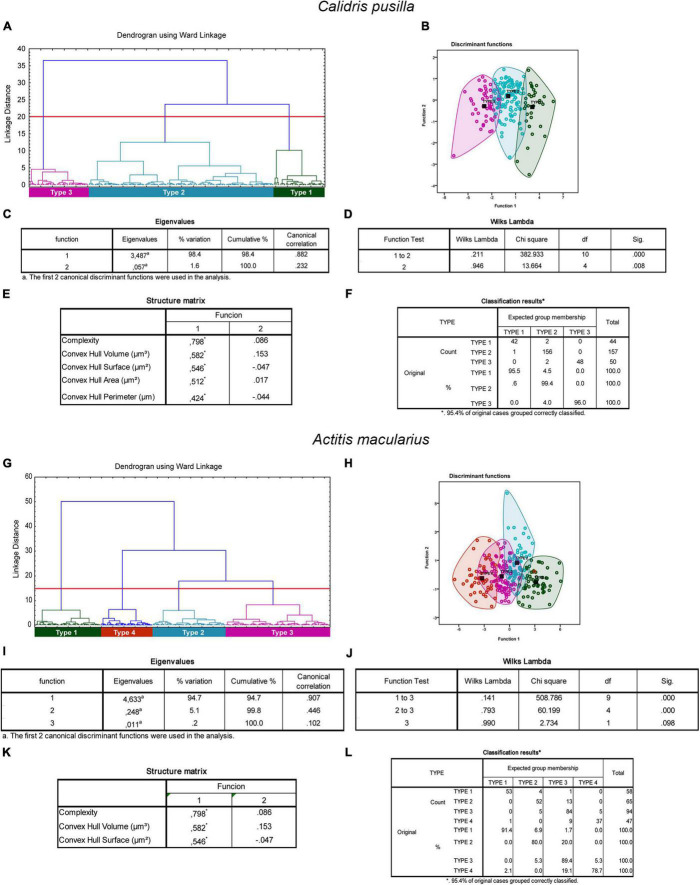
Cluster analysis of the morphology of astrocytes from rostral, intermediate, and caudal regions of the hippocampal formation of *Calidris pusilla* **(A–F)** and *Actitis macularius* **(G–L)**. Dendrogram representation of the hierarchical cluster analysis (Ward’s method) of the 251 cells of five individual *C. pusilla* **(A)**. Notice three main morphological phenotypes named Type I (green), Type II (blue), and Type III (magenta). Graphic representation of the canonical discriminant function analysis illustrates the distribution of the three main clusters of astrocytes in the Euclidean space **(B)**. Colored circles green, blue, and magenta identify individual astrocytes of Type I, Type II, and Type III morphotypes. The variables that most contributed to the cluster’s formation were morphological complexity and convex hull volume. The eigenvalues output **(C)** indicates that function 1 explains 98.4% of the variance. Wilks’s lambda values in the function test 1–2, rejected the null hypothesis of no differences between the groups for these 2 variables (0.211; *p* = 0.000). In the structure matrix panel **(E)** is displayed the relative contributions of the variables to the cluster formation of functions 1 and 2. Classification results for the expected group membership **(F)**. Dendrogram representation of hierarchical cluster analysis (Ward’s method) of 264 reconstructed astrocytes of *Actitis macularius* (*n* = 5) **(G)**. Four main morphological phenotypes named Type I (green), Type II (blue), Type III (magenta), and Type 4 (orange) were found. Graphic representation of the canonical discriminant function analysis illustrates the distribution of the four main clusters of astrocytes in the Euclidean space **(H)**. Colored circles green, blue, magenta, and orange identify individual astrocytes of Type I, Type II, Type III, and Type IV morphotypes respectively. The variables that most contributed to the cluster’s formation were morphological complexity and convex hull volume. The eigenvalues output (I) indicates that function 1 explains 94.7%% of the variance. Wilks’s lambda values in the function test 1–3, and 2–3 **(J)**, rejected the null hypothesis of no differences between the classified groups (Wilks Lambda = 0.141 and 0.793; *p* = 0.000). In the structure matrix panel **(K)** is displayed the relative contributions of the variables to the cluster formation of functions 1 and 2. Classification results for the expected group membership **(L)**. Black squared dots indicate de centroid of each cluster. (*) indicates statistically significant difference.

In migratory species, the morphological complexity, volume, surface, area, and perimeter of the convex hull were the multimodal variables that contributed most to the cluster formation in the different species (Figures 5, 6). The morphological complexity and volume of the convex hull were the morphometric features that contributed most for cluster formation in all species (Figures 5I,J, 6C,D,I,J). Function 1 explained 77.1, 98.4, and 94.7% of the sample variance in the species *C. semipalmatus*, *C. pusilla*, and *A. macularius*, respectively (Figures 5C,D). Wilks’s lambda value correspondent to 1 and 2 canonical discriminant functions was the most important for groups separation in the species *C. semipalmatus* and *C. pusilla* (Wilks lambda: *C. semipalmatus* = 0.185; *p* = 0.000; *C. pusilla* = 0.211; *p* = 0.000) (Figures 5K, 6E) and Wilks’s lambda value correspondent to 1 and 3 canonical discriminant functions was the most important for groups separation in *A. macularius* (Wilks lambda = 0.141; *p* = 0.000) (Figure 6K) with 95.4% of the group data correctly classified in the three species (Figures 5H, 6F,H).

**FIGURE 7:**
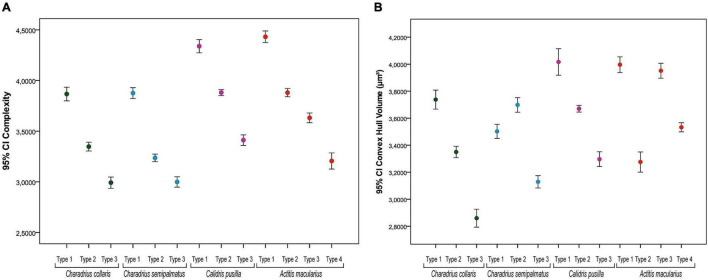
Mean values of morphological complexity **(A)** and of the convex hull volume **(B)** of astrocytes from the hippocampal formation of migratory (*C. semipalmatus* = blue, *C. pusilla* = magenta, and *A. macularius* = orange) and non-migratory (*C. collaris* = green dots) bird species and their confidence intervals (CI). Except for *Actitis macularius* which shows four groups of morphotypes (types I–IV) the dots indicate mean values for three morphotypes (types I–III). Whiskers show 95% CI. All species showed statistically significant differences in the mean values of morphological complexity for all comparisons between morphotypes. Intervals that do not overlap represent statistically significant differences (*p* < 0.05).

After evaluation of the data to select multimodal variables for hierarchical cluster analysis using variance-shrunk (logarithmized) parameters, we identified the groups in the dendrogram with statistically significant differences, followed by canonical discriminant analysis (Wiltschko W. and Wiltschko, 2012) (see, Figures 5–7). The canonical discriminant function analysis performed very well demonstrating its ability to predict astrocyte morphology, with high classification accuracy of individuals in the three different groups in *C. collaris* (97.4% for type I, 98.3% for type II and 87.5% for type III), and *C. semipalmatus* (95% for type I, 90% for type II and 96.7% for type III) and *C. pusilla* (95.5% for type I, 99.4% for type II and 96% for type III) and in four different groups in *A. macularius* (91.4% for type I, 80% for type 2, 89.4% for type III and 78.7% for type IV), confirming the existence of three distinct groups in *C. collaris*, *C. semipalmatus*, and *C. pusilla* and four groups in *A. macularius*. See Figures 5, 6 for details.

The comparative analysis between species, with all morphotypes revealed significant differences in all comparisons for morphological complexity [PERMANOVA: *C. collaris*—*F* (3;260) = 190.33, *p* = 0.0001; *C. semipalmatus*—*F* (3;302) = 226.65, *p* = 0.0001; *C. pusilla*—*F* (3;251) = 164.06, *p* = 0.0001; *A. macularius*—*F* (4;264) = 216.8, *p* = 0.0001] and significant differences in pairwise comparisons within all species (*p* = 0.0001) (Supplementary Tables 8–11).

Figure 7 is a graphic representation for these findings of morphological complexity and convex hull volume. As mentioned before these are the variables that contributed most to cluster formation in all species. It is important to highlight that there was no linear correspondence between morphotype mean values of convex hull volume in *C. pusilla* (with three morphotypes) and *A. macularius* (with four morphotypes) in the graphic representation of this variable in Figure 7.

**FIGURE 8:**
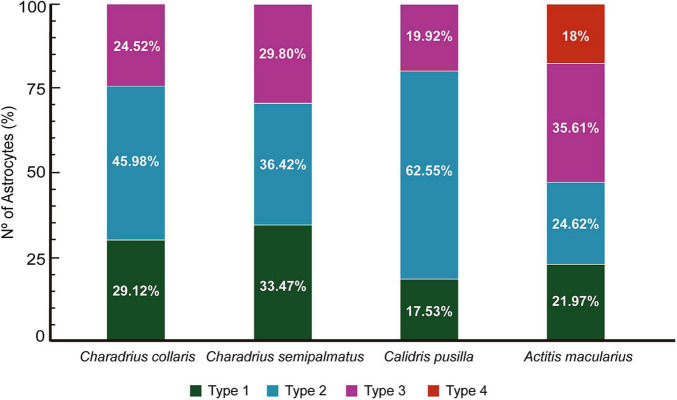
Relative percentage of Type I, Type II, Type III and Type IV astrocytes in the hippocampal formation of *C. collaris*, *C. semipalmatus*, *C. pusilla*, and *A. macularius*. Note that except by *A. macularius* where type III appears in greater proportion, astrocytes of intermediate morphological complexity (Type II) are more frequent in all other three species. Please remember that we named astrocytes based on morphological complexity as previously described in shorebirds (Carvalho-Paulo et al., 2018; da Costa et al., 2020; Henrique et al., 2020). Type I designates the morphotype with greater morphological complexity mean value in all species and that a progressive reduction in the mean values of morphological complexity is observed from type I to type IV.

The percent distribution of each morphotype in each species is shown in Figure 8. The semipalmated sandpiper *C. pusilla* had a higher percentage of type II (62.55%) than type I (17.53%) or type III (19.92%), and these values contrast with the percentual distributions of type II, type I, and type III astrocytes of *C. semipalmatus* (36.42, vs 33.47 vs 29.8%), *A. macularius* (24.62 vs 21.97 vs 35.61 vs 18% of type IV), or *C. collaris* (45.98 vs 29.12 vs 24.52%) (Figure 8). Thus, except for *A. macularius* where type III showed higher frequency, astrocytes of intermediate morphological complexity (Type II morphotype) are more frequent in all other species. Please remember that Type I designates the morphotype with greater morphological complexity mean value in all species.

Permutational analysis of multivariate dispersions of 17 morphometric features showed significant interaction between species and astrocyte type [PERMIDISP (7,1069) *F*: 5,277; *p* = 0.0001). In the paired tests for species there were differences in dispersion for almost all species (*p* < 0.05), except between non-migratory *C. collaris* and the species with the longest migratory distance *A. macularius* (*t* = 0.711; *p* = 0.477). Considering only the factor “type,” however, there was no significant dispersion (type I × type II; *t* = 0.973; *p* = 0.338) (see Supplementary Table 12).

Regarding the differences in Euclidean space, we found significant differences in the interaction of the factors [PERMANOVA; *F* (3.1069) = 5,479; *p* = 0.0001], “species” [*F* (3.1069) = 106.82; *p* = 0.0001] and “type” [*F* (3.1069) = 186.69; *p* = 0.0001]. All pairwise comparisons showed significant differences, including comparisons between species, between types and interactions between the two factors (p < 0.001). Thus, when we analyze the results of PERMIDISP and PERMANOVA, we can say that only the factor “type” showed differences in the location of the samples in the Euclidean space. Regarding the factors “species and “species × type” the differences found were due to the dispersion of the samples.

**FIGURE 9:**
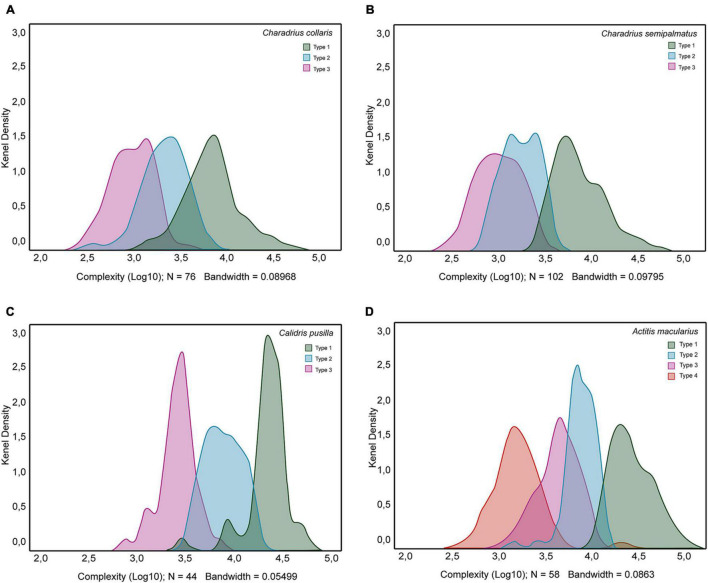
Kernel density plots to display where values of morphological complexity are concentrated over the interval covered by the dataset of *Charadrius collaris* **(A)**, *Charadrius semipalmatus* **(B)**, *Calidris pusilla* **(C)**, and *Actitis macularius* **(D)**. Notice that except by *A. macularius* where we found four peaks, in all other density plots we identified three peaks. Green, blue, magenta, and orange colors identify distribution of morphological complexity values of morphotypes I, II, III. and IV, respectively.

Figure 9 exhibits a Kernel density plot of the distribution of morphological complexity values of distinct morphotypes of hippocampal astrocytes from the dataset of *C. collaris*, *C. semipalmatus*, *C. pusilla*, and *A. macularius*. As expected, except for *A. macularius* where we identified four peaks, all other species showed three peaks, confirming that morphological complexity can be used in isolation to distinguish the morphotypes of astrocytes of the hippocampal formation.

**FIGURE 10:**
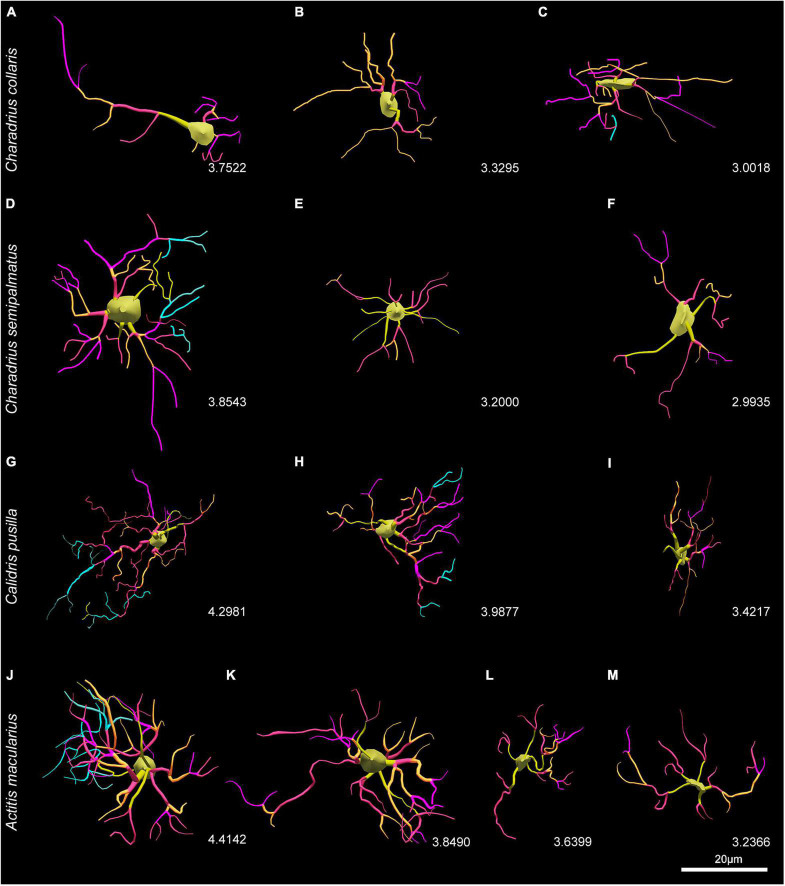
Three-dimensional reconstructions of representative cells of *C. collaris* **(A–C)**, *C. semipalmatus* **(D–F)**, *C. pusilla* **(G–I)**, and *A. macularius* **(J–M)** indicating correspondent logarithmized values of morphological complexity of each morphotype. Note that the values of the decimal logarithm of the morphological complexity of the representative mean cell of each morphotype are displayed for comparison purposes between the species. Scale bars = 25 μm.

Another way of visualizing the influence of migratory behavior on the morphology of astrocytes is to observe the representative 3D mean cells of each group (Figure 10). While in *A. macularius* we named type IV the hippocampal astrocytes with the lowest complexity, in species with three morphotypes (*C. collaris*, *C. semipalmatus*, and *C. pusilla*) the lowest complexity astrocytes corresponded to type III. Notice the greater morphological complexity mean values of hippocampal astrocytes in migratory species as compared to the non-migratory *C. collaris*.

**FIGURE 11:**
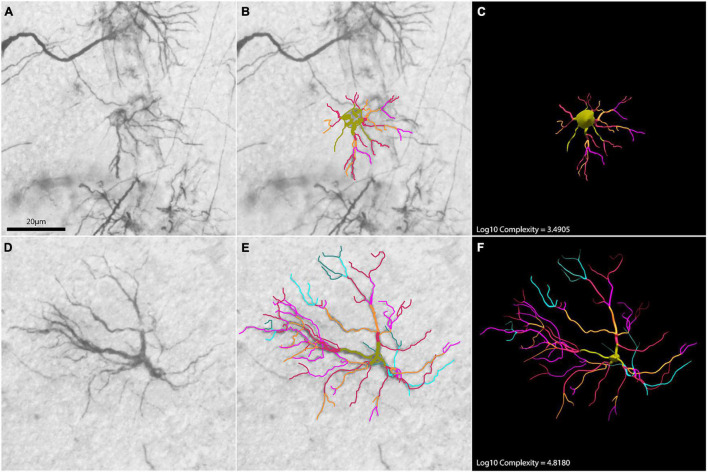
Two astrocyte photomicrographs **(A,D)** overlayed with correspondent branch reconstructions **(B,E)** are used to illustrate contrasting morphological complexity values. Panels **(C,F)** are correspondent 3D reconstructions of astrocytes illustrating low and high morphological complexities, respectively. Numbers indicate correspondent logarithmized values of morphological complexity.

Two raw astrocytes images overlayed with branch reconstructions used to illustrate contrasting morphological values are exhibited on Figure 11. They are examples of higher and lower morphological complexity astrocytes from the hippocampal formation of shorebirds which were microscopically reconstructed in 3D.

## Discussion

The systematic search for correlations between genes and behavioral phenotypes, looking at differential gene expression or the occurrence of allele polymorphisms associated with a specific phenotype, may be useful for understanding the mechanisms underlying the migration process and its molecular control (Bazzi et al., 2016b; Contina et al., 2018). Although the observed relationships between candidate genes *CLOCK* and *ADCYAP1* and migratory behavior in birds appear to vary across species, previous studies revealed that both loci can be significantly correlated with a variety of distinct phenotypes in long distance migratory birds (Saino et al., 2015; Bazzi et al., 2016b). For example, in blackpoll warblers (*Setophaga striata*), a Neotropical-Nearctic migrant, minimum allele length was associated with later spring departure date for breeding grounds and earlier fall arrival date at wintering ground (Ralston et al., 2019).

The *ADCYAP1* gene codes for PACAP which influences circadian rhythms directly by activating *CLOCK* and other genes in the circadian oscillator complex (Nagy and Csernus, 2007). PACAP also modulates a number of astrocyte activities such as proliferation, plasticity, glycogen production, and biosynthesis of neurotrophic factors and gliotransmitters (Masmoudi-Kouki et al., 2007). In previous studies we demonstrated that contrasting long-distance migratory flights of *C. pusilla* and *C. semipalmatus* differentially shape the morphological complexity of two morphotypes of hippocampal astrocytes (Henrique et al., 2020). Here we searched for associations between astrocyte morphological complexity, migratory distance, and size of the *ADCYAP1* allele. Our findings showed significant differences in the size of the microsatellites of *C. pusilla*, *C. semipalmatus*, *A. macularius*, and *C. collaris*, in association with distinct mean values of astrocyte morphological complexity in the hippocampal formation of these species. These findings support previous suggestions for a role of *ADCYAP1* in shaping the avian migratory phenotype (Bazzi et al., 2016b) and its relation with astroglial physiology. We propose that these associations may be part of the adaptive response to the migratory process.

### The Increase in *ADCYAP1* Microsatellite Repetitions Was Associated With Longer Distances Migration

Before and during migration, birds undergo significant changes in physiology and behavior that are adapted and motivated differently in spring and autumn: spring migration takes place in search of reproductive sites while autumn migration takes place in search of wintering sites with milder temperatures and greater food availability (Sharma et al., 2018a,b). As compared with autumn migration, buntings show greater body mass, higher levels of triglycerides and free fatty acids, accumulate more subcutaneous fat and liver lipids, and more intense Zugunruhe in spring migration (Samojłowicz et al., 2019). A relação entre o tamanho do microsatellite e diferentes funções fisiológicas foi previamente descrita (Ralston et al., 2019). In the present report all individuals were captured between August 15 and April 8, during the wintering period in the mangroves of Bragança (Brazil) in the Amazon River estuary, where they experienced temperatures between 20.4 and 32.8°C with the minimum values coincident with the increase in pluviometry mean values in March (Ribeiro, 2001).

In all comparisons of mean allele length between migrant species (*A. macularius*, *C. pusilla*, and *C. semipalmatus*), and the non-migratory species (*C. collaris*) we found significant differences, with the lower mean value for Euclidean distance found in the non-migratory species. In addition, the mean values of allele length in migratory birds increased significantly as a function of migratory distance.

Thus, based on earlier studies of *ADCYAP1* variation (Mueller et al., 2011), our prediction that migratory species *A. macularius*, *C. pusilla*, and *C. semipalmatus* would possess longer microsatellite repeat-length alleles of *ADCYAP1* compared with those of non-migratory *C. collaris*, was confirmed. In addition, we found proportional direct association between the size of microsatellite repeat-length alleles at the *ADCYAP1* locus and autumnal migratory distances. These findings are in line with previous studies that demonstrated that *ADCYAP1* polymorphism covaries with breeding latitude (Bazzi et al., 2016b).

### The Morphological Complexity of Astrocytes Is Greater in Migrating Birds

Differences in migratory behavior affect not only the number of astrocytes in the hippocampal formation of migrating birds, but also their morphology (Henrique et al., 2020). Indeed, Henrique and collaborators compared the number and 3-D morphology of hippocampal astrocytes of *C. semipalmatus* before and after autumnal migration with those of *C. pusilla* to test the hypothesis that the contrasting migratory flights of these species could differentially shape hippocampal astrocyte number and morphology. *C. pusilla* migration to the southern hemisphere includes a 5-day non-stop flight over the Atlantic Ocean (Brown, 2014), whereas *C. semipalmatus* migration, to the same area, is largely over land with stopovers for feeding and rest. After hierarchical cluster analysis of astrocyte morphological features, two families of morphological phenotypes in *C. pusilla* and *C. semipalmatus* hippocampal formations named Type I and Type II were distinguished, which were differentially affected after autumnal migratory flights. Stereological counts of hippocampal astrocytes demonstrated that the number of astrocytes decreased significantly in *C. pusilla* but did not change in *C. semipalmatus*.

Thus, after hierarchical cluster analysis with non-normalized data, we previously found at the greater Euclidian distances two morphotypes of hippocampal astrocytes (Carvalho-Paulo et al., 2018; Henrique et al., 2020). Here, we performed hierarchical clustering on variance-shrunk logarithmized values of multimodal morphometrical features for reduction of the asymmetrical influence of non-standardized morphological features on cluster segregation. With logarithmized morphometric values, we compared the morphologies of astrocytes from the hippocampus of long-distance migratory birds with contrasting migratory flights (*C. semipalmatus*, *C. pusilla*, and *A. macularius*) with each other, and with a non-migratory species (*C. collaris*). Except for the hippocampal formation of *A. macularius* where we found four morphotypes, all other species showed three hippocampal astrocytes morphotypes. Thus, in this work, we expanded the studies of morphometry of astrocytes to *C. collaris* and *A. macularius* hippocampal formations indicating the occurrence of three main morphological types of astrocytes in *C. pusilla*, *C. semipalmatus*, and *C. collaris* and 4 morphotypes in *A. macularius*. Morphological complexity and convex hull volume were the variables that best distinguished the morphological families suggested by hierarchical cluster analysis. These morphological families of astrocytes were validated by comparing the mean values of morphological variables for type and species studied using PERMANOVA and canonical discriminant analysis. Type I was used to name the cell population with the highest average values of morphological complexity. Type II, Type III, and Type IV named astrocytes with decreased morphological complexity. We suggest that the differences found between migratory birds’ hippocampal astrocyte morphologies might be related to the adaptive response imposed by the contrasting long-distance migratory flights on learning and memory for recognition of olfactory, geomagnetic, and visual cues during migration or to recognize local cues for both migratory birds and non-migratory *C. collaris* (Mouritsen et al., 2016; Magalhaes et al., 2017; Carvalho-Paulo et al., 2018; da Costa et al., 2020; Henrique et al., 2020; Guerreiro et al., 2021).

Notably, a phylogenetically independent contrast (PIC) approach (Felsenstein, 1985) showed that most morphometric differences in astrocytes found in the different species were not influenced by phylogenetic differences (Henrique et al., 2020). The PIC approach used DNA sequences of intron 7 of beta-fibrinogen (fib 7) gene, recombination activating gene 1(RAG1), cytochrome oxidase c subunit 1 (COI) gene and cytochrome b (cyt *b*) gene obtained from GeneBank for *C. pusilla*, *C. semipalmatus, A. macularius*, and *C. collaris* (Henrique et al., 2020).

In the present report, however, we were expecting that *A. macularius* and *C. semipalmatus*, that rely more on remembering visual cues during overland migration than *C. pusilla*, which migrates via a long-distance non-stop flight over the Atlantic Ocean (Diniz et al., 2016) would show higher mean values of morphological complexity. We also hypothesized that *C. collaris*, a non-migrant species, as compared with the migrant species would show the lowest morphological complexity. We found that the non-migratory species *C. collaris* and *C. semipalmatus* showed similar mean values for type I (higher complexity) and III (lower complexity) and that type II (median complexity) mean values in *C. collaris* were greater than that of *C. semipalmatus*. Similar mean values were found for morphological complexity of type I and II in *A. macularius* and *C. pusilla* but these values were significantly higher than those of correspondently named astrocytes of *C. collaris* and *C. semipalmatus*. In addition, *A. macularius* showed greater type III mean values than those of *C. pusilla* correspondent morphotype.

From these findings, it is not possible to anticipate the mechanisms underneath the hippocampal astrocyte morphological differences between wintering migrating birds with contrasting migratory flights and non-migrating birds. However, in a previous study it clearly emerged that the glial morphologies of birds collected in August in the Bay of Fundy (Canada) and September to March on Isla Canela (Bragança, Brazil) are clearly different, both in *C. pusilla* and *C. semipalmatus* (Henrique et al., 2020). These findings were interpreted as due to contrasting migratory routes (non-stop transatlantic flight vs mostly overland migration). However, many other possibilities were pointed out as possible contributors to the morphological differences. For example, different capture dates (Carvalho-Paulo et al., 2018) with differential implications for hormones and receptors for stress and pre- and post-breeding conditions (Riou et al., 2010; Eikenaar et al., 2015; Deviche et al., 2016; Surbhi Rastogi et al., 2016). Here, we also raise the hypothesis that distinct morphotypes, may have differential physiological roles in different species and this may be at least part of the underlying hippocampal circuitry adaptive response for behavioral changes in those species (Hwang et al., 2021).

### Migratory Behavior, *ADCYAP1* Microsatellites and Hippocampal Astrocyte Morphology

*Calidris pusilla*, *C. semipalmatus*, and *A. macularius* migratory journeys between the northern breeding sites of United States and Canada and wintering grounds in the Amazon basin of northern South America are among the longest migratory routes of shorebirds. As previously indicated, significant association was observed between the size of the simple sequence repeats (SSR) and migration distance in these three migrant species. Consistent with this, differences in food intake and glucocorticoid effects during overland and transatlantic flights may differentially affect migrating and wintering birds’ metabolic pathways, with significant influences on astrocyte morphologies. For example, during the 5-day non-stop flight of *C. pusilla* a short supply of glucose and a high demand for lipids occurs, inducing the brain to increase ketone body metabolism to support the transoceanic flight (Achanta and Rae, 2017). This uninterrupted flight of *C. pusilla* compared with the multiple stopover flights of *C. semipalmatus* and *A. macularius*, may impose differential demand for PACAP, which is synthesized by *ADCYAP1*.

Because SSR in the regulatory region 3’UTR of *ADCYAP1* may modify gene function and post-transcriptional processes (Riley and Krieger, 2009; Steinmeyer et al., 2009) it is reasonable to expect that any changes in SSR found in *A. macularius*, *C. pusilla*, and *C. semipalmatus*, may benefit migratory behavior of these species. In agreement with this expectation, it has been demonstrated that in the CNS, PACAP is involved in the rhythmicity of melatonin production and in the increase of cAMP in birds (Nakahara et al., 2002; Nowak and Zawilska, 2003), as well as acting as a co-transmitter with glutamate to shift the phase of the CNS circadian rhythm in a similar way to light (Michel et al., 2006; Vaudry et al., 2009). In addition, previous data show that PACAP plays important role in controlling astroglial functions by regulating cell proliferation and glycogen metabolism (Magistretti et al., 1998; Masmoudi-Kouki et al., 2007; Nakamachi et al., 2011).

Moreover, PACAP is affected by diet and fasting (Iwasa et al., 2016; Nakata et al., 2016) with important implications for regulation of food intake (Kataoka et al., 2013; Nguyen et al., 2020) and energy homeostasis (Chang et al., 2021) and this seems to include birds (Tachibana et al., 2015; Simon et al., 2017). PACAP is also affected by photoperiodic light changes (Saino et al., 2015; Bazzi et al., 2016a; Adamska et al., 2018; Haraguchi et al., 2019), reproductive conditions (Prisco et al., 2019; Winters and Moore, 2020) and stress (Agarwal et al., 2005; Norrholm et al., 2005; Hammack et al., 2010). Due to differential pre- and post-breeding physiological conditions, distinct metabolic demands and diets imposed by migration with multiple stopovers and fasting uninterrupted transoceanic flight, along with differential stress levels along the migratory journey of migrant species compared to the non-migrant *C. collaris*, it may be possible that at least part of the astrocyte morphological differences between species may be associated with interspecific differential expression of *ADCYAP1*. Indeed, PACAP mimicked effects of forskolin, a direct activator of adenylate cyclase, on the actin cytoskeleton of astrocytes with resultant astrocyte morphological changes (Perez et al., 2005). In addition, PACAP is essential for lactate production and secretion in astrocytes, a central step in the neuronal physiology of hippocampal learning and memory (Kambe et al., 2021) the center for integrative information for familiar landmarks and landscape features in homing pigeons (Bingman and Ewry, 2020; Gagliardo et al., 2021), and for avian long-distance migratory journeys (Frost and Mouritsen, 2006; Barkan et al., 2016; Bingman and MacDougall-Shackleton, 2017).

Migratory phenotypes and *ADCYAP1* and *CLOCK* genes have been tested in other species including migratory distance, wing morphology and spring migration arrival of *Sylvia atricapilla* (Mueller et al., 2011; Mettler et al., 2015); migratory restlessness in the songbird genus *Junco* and *S. atricapilla* (Mueller et al., 2011; Peterson et al., 2013); time of *C. pusilla* migration (Bazzi et al., 2016b); and activation of *CLOCK* and other circadian genes in *Gallus gallus* (Nagy and Csernus, 2007).

In line with these findings, we found significant differences in all comparisons of allele mean length between migrant species (*A. macularius*, *C. pusilla*, and *C. semipalmatus*), and the non-migratory species (*C. collaris*), with the lower mean value in the non-migratory *C. collaris*. This may suggest that different migratory behaviors are associated with size differences of *ADCYAP1* microsatellites, and this may be acting through PACAP to induce morphological and functional changes in the astrocytes of the hippocampal formation (Vaudry et al., 2009). It is reasonable to assume as well, that in *C. collaris*, *ADCYAP1* microsatellites are not under positive selection for migratory behavior.

### Methodological Limitations and Potential Sources of Non-biological Variation

Because we did not track individuals during the migratory flights we estimated the distance between breeding sites and the wintering places of capture (see section “Materials and Methods”). We followed previous descriptions of suggested trajectories between stop overs for each species (Williams and Williams, 1978; Brown, 2014; Anderson et al., 2019), as indicated in Figure 2. Because *C. collaris* is not a migrant species we arbitrarily attributed 0 km as distance traveled for this species and because this species is not sedentary, this is a limitation of the present report. Migrant birds also move during the wintering periods and their local movements were ignored as well. Because local displacements of all individuals during the wintering period may not be similar in different species, their potential influence on astrocytes morphologies could not be assessed, and this is limitation to be explored in future studies.

To measure possible influence of capture dates on hippocampal astrocyte morphological complexities of wintering birds, in a previous report dedicated to *C. pusilla*, we compared astrocyte morphologies of birds captured at different time points of the wintering period using the same statistical analysis (Carvalho-Paulo et al., 2018). We found smaller changes in the mean values of hippocampal astrocytes morphological complexity which did not significantly affect the results (Carvalho-Paulo et al., 2018). However, recent findings from morphological analysis of hippocampal astrocytes of Arenaria interpres, differed significantly in the morphological complexity of hippocampal astrocytes of autumnal recently arrived migrant birds (captured in September/October) and spring premigratory individuals (captured in April/May) suggesting that as wintering period progresses, significant changes in hippocampal circuitry occur (da Costa et al., 2020). Thus, it is reasonable to expect that astrocytes have their morphology changed during the wintering period.

Because there is no information in the literature about potential influence of sex and age on hippocampal astrocyte morphology in long-distance migratory birds, and we did not measure the age of individuals in our sample due to technical limitations, it is difficult to discuss these potential influences in detail. However, experience and sex are important variables that have been previously demonstrated to influence hippocampal-dependent tasks in birds (Astié et al., 2015; Rensel et al., 2015; Guigueno et al., 2016; Bingman and MacDougall-Shackleton, 2017), and migratory behavior is accompanied by hippocampal morphological changes including volume, and neurogenesis (Barkan et al., 2016, 2017; de Morais Magalhaes et al., 2017) which should be considered in future studies of hippocampal astrocyte morphologies in long-distance migratory birds.

Although the correlational analysis between the length of *ADCYAP1* microsatellites, distance of migration, and differences in the morphologies of astrocytes of the hippocampal formation seems to be coherent, the story could be different if another higher order brain area, less involved in migration, exhibited similar astrocytes differences and this is a potential limitation of the present study, that could be avoided if another area was explored (Carvalho-Paulo et al., 2018; Henrique et al., 2020).

It is not uncommon to find contradictory results in comparative studies due to ambiguities in the definition of the objects and areas of interest, variations in histological procedures, or in the case of 3D reconstructions, dissimilarities in the sampling approach to select cells for reconstruction (West, 2002). In this report all samples were obtained with the same tissue processing protocols (perfusion, antigen retrieval, immunoreaction, dehydration, counterstaining, and clearing) and the specificity of the immunohistochemical pattern was confirmed using a control reaction that omitted the primary antibody (Saper and Sawchenko, 2003; Schmitt et al., 2004; Fritschy, 2008). To obtain sufficient contrast between foreground and background we improved the signal/noise ratio with glucose-oxidase-DAB-Nickel peroxidase amplification method (Shu et al., 1988).

It has been also demonstrated that the z-axis (perpendicular to the cutting surface), shrinks by approximately 75% of the cut thickness after dehydration and clearing (Carlo and Stevens, 2011). Based on those findings, all astrocytes’ reconstructions were corrected for z-axis shrinkage. No corrections were applied to X/Y axes and used the same software and hardware approaches for sampling, reconstruction, and analysis. These procedures guarantee similar, systematic and random sampling selection of astrocytes across all regions of the areas of interest. Finally, to detect possible variations in the criteria for identifying and including only complete astrocyte arbors inside the area of interest, we undertook checking procedures of the results by having different investigators reconstruct astrocytes in the same regions using the same GFAP antibody as a marker for selective labeling. Thus, we expected to reduce possible sources of non-biological variation.

## Conclusion

The largest microsatellite repeat-length alleles, the highest mean value of astrocyte morphological complexity and the longest migratory distance were found in *A. macularius*, followed by intermediate values in *C. pusilla* and *C. semipalmatus*, while the smallest microsatellite repeat-length alleles and the smallest morphological complexity mean values were found in the resident non-migratory *C. collaris*. Taking these findings together, we suggest that polymorphism in the gene *ADCYAP1* may underlie variation in the migratory phenotype and both are strongly related to migratory distances.

## Data Availability Statement

The original contributions presented in the study are included in the article/Supplementary Material, further inquiries can be directed to the corresponding author. Tabulated data used for microsatellite sizes and astrocyte morphological parameters are available from: https://github.com/patrick-douglas/Miranda_et_al_2021.

## Ethics Statement

The animal study was reviewed and approved under license N° 44551-2 from the Chico Mendes Institute for conservation of Biodiversity (ICMBio) and Scientific Capture permit ST2783 from the Canadian Wildlife Service. All procedures were carried out in accordance with the Association for the Study of Animal Behavior / Animal Behavior Society Guidelines for the Use of Animals in Research and with approval of the Animal Users Subcommittee of the University of Western Ontario. All efforts were made to minimize the number of animals used, stress and discomfort.

## Author Contributions

All authors contributed substantially to the conception or design of the work, acquisition, analysis, or interpretation of data for the work, drafting the work or revising it critically for important intellectual content, and/or final approval of the version to be published, and agreed to be accountable for all aspects of the work in ensuring that questions related to the accuracy or integrity of any part of the work are appropriately investigated and resolved.

## Conflict of Interest

The authors declare that the research was conducted in the absence of any commercial or financial relationships that could be construed as a potential conflict of interest.

## Publisher’s Note

All claims expressed in this article are solely those of the authors and do not necessarily represent those of their affiliated organizations, or those of the publisher, the editors and the reviewers. Any product that may be evaluated in this article, or claim that may be made by its manufacturer, is not guaranteed or endorsed by the publisher.

## Abbreviations

3-D: three dimensional reconstructions
ABC: avidin–biotin–peroxidase complex
*ADCYAP1*: adenylate cyclase activating polypeptide 1
ANOVA: analysis of variance
CE: coefficient of error
CNS: central nervous system
CV: coefficient of variation
CVB: coefficient of biological variation
DAB: diaminobenzidine
DNA: deoxyribonucleic acid
FH: hippocampal formation
GFAP: glial fibrillary acid protein
GLM: general linear model
ICMBio: Chico Mendes Institute for Biodiversity Conservation
M3: asymmetry
M4: = kurtosis
MMI: multimodality index
PACAP: pituitary adenylate cyclase-activating polypeptide
PBS: phosphate-buffered saline
PBST: Triton ™ phosphate-buffered saline
PCO: principal coordinate analysis
PCR: polymerase chain reaction
PERMANOVA: analysis of variance by multivariate permutation
PERMIDISP: dispersion homogeneity tests
SE: standard error
UTR: untranslated region.

## References

[B1] Able K. P. (1991). The development of migratory orientation mechanisms. *EXS* 60 166–179. 10.1007/978-3-0348-7208-9_8 1838514

[B2] Achanta L. B. Rae C. D. (2017). β-hydroxybutyrate in the brain: one molecule. Multiple Mechanisms. *Neurochem. Res.* 42 35–49. 10.1007/s11064-016-2099-2 27826689

[B3] Adamska I. Malz M. Lewczuk B. Blügental N. Markowska M. A. Meronka R. (2018). Daily profiles of neuropeptides, catecholamines, and neurotransmitter receptors in the chicken pineal gland. *Front. Physiol.* 9:1972. 10.3389/fphys.2018.01972 30697171 PMC6340997

[B4] Agarwal A. Halvorson L. M. Legradi G. (2005). Pituitary adenylate cyclase-activating polypeptide (PACAP) mimics neuroendocrine and behavioral manifestations of stress: Evidence for PKA-mediated expression of the corticotropin-releasing hormone (CRH) gene. *Brain Res. Mol. Brain Res.* 138 45–57. 10.1016/j.molbrainres.2005.03.016 15882914 PMC1950324

[B5] Åkesson S. Ilieva M. Karagicheva J. Rakhimberdiev E. Tomotani B. Helm B. (2017). Timing avian long-distance migration: from internal clock mechanisms to global flights. *Philos. Trans R Soc. Lond. B Biol. Sci.* 372:20160252. 10.1098/rstb.2016.0252 28993496 PMC5647279

[B6] Anderson A. Duijins S. Smith P. A. Friis C. Nol E. (2019). Migration distance and body condition influence shorebird migration sttrategies and stopover decisions during southband migration. *Front. Ecol. Evol.* 7:251.

[B7] Anderson M. Ellingsen K. McArdle B. (2006). Multivariate dispersion as a measure of beta diversity. *Ecol. Lett.* 9 683–693. 10.1111/j.1461-0248.2006.00926.x 16706913

[B8] Anderson M. Gorley R. Clarke K. (2008). *For PRIMER: Guide to Software and Statistical Methods.* Plymouth: Prim.Plymouth.

[B9] Astié A. A. Scardamaglia R. C. Muzio R. N. Reboreda J. C. (2015). Sex differences in retention after a visual or a spatial discrimination learning task in brood parasitic shiny cowbirds. *Behav. Proc.* 119 99–104. 10.1016/j.beproc.2015.07.016 26248015

[B10] Atoji Y. Wild J. M. (2004). Fiber connections of the hippocampal formation and septum and subdivisions of the hippocampal formation in the pigeon as revealed by tract tracing and kainic acid lesions. *J. Comp. Neurol.* 475 426–461. 10.1002/cne.20186 15221956

[B11] Atoji Y. Sarkar S. Wild J. M. (2016). Proposed homology of the dorsomedial subdivision and V-shaped layer of the avian hippocampus to Ammon’s horn and dentate gyrus, respectively. *Hippocampus* 26 1608–1617. 10.1002/hipo.22660 27657725

[B12] Bakalar D. Sweat S. Drossel G. Jiang S. Z. Samal B. B. Stroth N. (2021). Relationships between constitutive and acute gene regulation, and physiological and behavioral responses, mediated by the neuropeptide PACAP. *Psychoneuroendocrinology* 135:105447. 10.1016/j.psyneuen.2021.105447 PMC8900973 34741979

[B13] Baliga R. S. Macallister R. J. Hobbs A. J. (2013). Vasoactive peptides and the pathogenesis of pulmonary hypertension: role and potential therapeutic application. *Handb. Exp. Pharmacol.* 218 477–511. 10.1007/978-3-642-38664-0_19 24092352

[B14] Balloux F. Lugon-Moulin N. (2002). The estimation of population differentiation with microsatellite markers. *Mol. Ecol.* 11 155–165. 10.1046/j.0962-1083.2001.01436.x 11856418

[B15] Barkan S. Roll U. Yom-Tov Y. Wassenaar L. I. Barnea A. (2016). Possible linkage between neuronal recruitment and flight distance in migratory birds. *Sci. Rep.* 6:21983. 10.1038/srep21983 26905978 PMC4764934

[B16] Barkan S. Yom-Tov Y. Barnea A. (2017). Exploring the relationship between brain plasticity, migratory lifestyle, and social structure in birds. *Front. Neurosci.* 11:139. 10.3389/fnins.2017.00139 28396621 PMC5367377

[B17] Barros J. Winkler F. M. Velasco L. A. (2020). Assessing the genetic diversity in. *Ecol. Evol.* 10 3919–3931. 32489620 10.1002/ece3.6080 PMC7244797

[B18] Bazzi G. Galimberti A. Hays Q. R. Bruni I. Cecere J. G. Gianfranceschi L. (2016b). Adcyap1 polymorphism covaries with breeding latitude in a nearctic migratory songbird, the Wilson’s warbler (*Cardellina pusilla*). *Ecol. Evol.* 6 3226–3239. 10.1002/ece3.2053 27252831 PMC4870208

[B19] Bazzi G. Cecere J. G. Caprioli M. Gatti E. Gianfranceschi L. Podofillini S. (2016a). Clock gene polymorphism, migratory behaviour and geographic distribution: a comparative study of trans-saharan migratory birds. *Mol. Ecol.* 25 6077–6091. 10.1111/mec.13913 27862517

[B20] Billerman S. Bk K. Pg P. Ts S. (eds) (2020). *Birds of the World.* Ithaca, NY: Cornell Laboratory of Ornithology.

[B21] Bingman V. P. Ewry E. M. (2020). On a search for a neurogenomics of cognitive processes supporting avian migration and navigation. *Integr. Comp. Biol.* 60 967–975. 10.1093/icb/icaa040 32426820

[B22] Bingman V. P. MacDougall-Shackleton S. A. (2017). The avian hippocampus and the hypothetical maps used by navigating migratory birds (with some reflection on compasses and migratory restlessness). *J. Comp. Physiol. A Neuroethol. Sens. Neural. Behav. Physiol.* 203 465–474. 10.1007/s00359-017-1161-0 28299428

[B23] Bornstein J. C. Costa M. Grider J. R. (2004). Enteric motor and interneuronal circuits controlling motility. *Neurogastroenterol Motil* 16(Suppl. 1) 34–38. 10.1111/j.1743-3150.2004.00472.x 15066002

[B24] Boutin P. Hani E. H. Vasseur F. Roche C. Bailleul B. Hager J. (1997). Automated fluorescence-based screening for mutation by SSCP: use of universal M13 dye primers for labeling and detection. *Biotechniques* 23 358–362. 10.2144/97233bm01 9298196

[B25] Boutin-Ganache I. Raposo M. Raymond M. Deschepper C. F. (2001). M13-tailed primers improve the readability and usability of microsatellite analyses performed with two different allele-sizing methods. *Biotechniques* 31:28. 11464515

[B26] Bozadjieva-Kramer N. Ross R. A. Johnson D. Q. Fenselau H. Haggerty D. L. Atwood B. (2021). The role of mediobasal hypothalamic PACAP in the control of body weight and metabolism. *Endocrinology* 162:bqab012. 10.1210/endocr/bqab012 33460433 PMC7875177

[B27] Brown S. (2014). *The Remarkable Odyssey of a Semipalmated Sandpiper. In Shorebird Science.* Canada: Manomet Soaring Solutions Grounded Science.

[B28] Campos C. Naiff R. Araujo A. (2008). Censo de Aves Migratórias (Charadriidae e Scolopacidae) da Porção Norte da Bacia Amazônica, Macapá, Amapá, Brasil. *Ornithologia* 3, 38–46.

[B29] Carlo C. N. Stevens C. F. (2011). Analysis of differential shrinkage in frozen brain sections and its implications for the use of guard zones in stereology. *J. Comp. Neurol.* 519 2803–2810. 10.1002/cne.22652 21491430

[B30] Carvalho-Paulo D. de Morais Magalhães N. G. de Almeida Miranda D. Diniz D. G. Henrique E. P. I Moraes A. M. (2017). Hippocampal astrocytes in migrating and wintering semipalmated sandpiper. *Front. Neuroanat* 11:126. 10.3389/fnana.2017.00126 PMC5758497 29354035

[B31] Carvalho-Paulo D. Magalhaes N. G. D. Miranda D. D. Diniz D. G. Henrique E. P. I Moraes A. M. (2018). Hippocampal astrocytes in migrating and wintering semipalmated sandpiper calidris pusilla. *Front. Neuroanatomy* 11:126. 10.3389/fnana.2017.00126 29354035 PMC5758497

[B32] Chang R. Hernandez J. Gastelum C. Guadagno K. Perez L. Wagner E. J. (2021). Pituitary adenylate cyclase-activating polypeptide excites proopiomelanocortin neurons: implications for the regulation of energy homeostasis. *Neuroendocrinology* 111 45–69. 10.1159/000506367 32028278

[B33] Ciranna L. Costa L. (2019). Pituitary adenylate cyclase-activating polypeptide modulates hippocampal synaptic transmission and plasticity: new therapeutic suggestions for fragile X syndrome. *Front. Cell Neurosci.* 13:524. 10.3389/fncel.2019.00524 31827422 PMC6890831

[B34] Clarke K. Warwick R. (2001). *Change in Marine Communities: An Approach to Statistical Analysis and Interpretation.* 2nd Edn. Plymouth: PRIMER-E, 172.

[B35] Contina A. Bridge E. S. Ross J. D. Shipley J. R. Kelly J. F. (2018). Examination of clock and adcyap1 gene variation in a neotropical migratory passerine. *PLoS One* 13:e0190859. 10.1371/journal.pone.0190859 29324772 PMC5764313

[B36] da Costa E. R. Henrique E. P. da Silva J. B. Pereira P. D. C. de Abreu C. C. Fernandes T. N. (2020). Changes in hippocampal astrocyte morphology of ruddy turnstone (arenaria interpres) during the wintering period at the mangroves of Amazon river estuary. *J. Chem. Neuroanat* 108:101805. 10.1016/j.jchemneu.2020.101805 32505650

[B37] de Morais Magalhaes N. G. Guerreiro Diniz C. Guerreiro Diniz D. Pereira Henrique E. Correa Pereira P. D. Matos Moraes I. A. (2017). Hippocampal neurogenesis and volume in migrating and wintering semipalmated sandpipers (*Calidris pusilla*). *PLoS One* 12:e0179134. 10.1371/journal.pone.0179134 28591201 PMC5462419

[B38] Del Hoyo J. Elliot A. Sargatal J. (1992). *Handbook of the Birds of the World.* Barcelona: Lynx Editions.

[B39] Dennis T. E. Rayner M. J. Walker M. M. (2007). Evidence that pigeons orient to geomagnetic intensity during homing. *Proc. Biol. Sci.* 274 1153–1158. 10.1098/rspb.2007.3768 17301015 PMC2189574

[B40] Deviche P. Valle S. Gao S. Davies S. Bittner S. Carpentier E. (2016). The seasonal glucocorticoid response of male rufous-winged sparrows to acute stress correlates with changes in plasma uric acid, but neither glucose nor testosterone. *Gen Comp. Endocrinol.* 235 78–88. 10.1016/j.ygcen.2016.06.011 27292791

[B41] Diniz C. G. Magalhães N. G. Sousa A. A. Santos Filho C. Diniz D. G. Lima C. M. (2016). Microglia and neurons in the hippocampus of migratory sandpipers. *Braz J. Med. Biol. Res.* 49:e5005. 10.1590/1414-431X20155005 26577847 PMC4678657

[B42] Eikenaar C. Müller F. Klinner T. Bairlein F. (2015). Baseline corticosterone levels are higher in migrating than sedentary common blackbirds in autumn, but not in spring. *Gen Comp. Endocrinol.* 224 121–125. 10.1016/j.ygcen.2015.07.003 26163918

[B43] Ellegren H. (2004). Microsatellites: simple sequences with complex evolution. *Nat. Rev. Genet.* 5 435–445. 10.1038/nrg1348 15153996

[B44] Emlen S. (1975). The stellar-orientation system of a migratory bird. *Sci. Am.* 233 102–111. 10.1038/scientificamerican0875-102 1145171

[B45] Excoffier L. Laval G. Schneider S. (2007). Arlequin (version 3.0): an integrated software package for population genetics data analysis. *Evol. Bioinform* 1 47–50. PMC2658868 19325852

[B46] Felsenstein J. (1985). Phylogenies and the comparative method. *Am. Nat.* 125 1–15. 10.1086/284325

[B47] Fritschy J. M. (2008). Is my antibody-staining specific? How to deal with pitfalls of immunohistochemistry. *Eur. J. Neurosci.* 28 2365–2370. 10.1111/j.1460-9568.2008.06552.x 19087167

[B48] Frost B. J. Mouritsen H. (2006). The neural mechanisms of long distance animal navigation. *Curr. Opin. Neurobiol.* 16 481–488. 10.1016/j.conb.2006.06.005 16839758

[B49] Gagliardo A. Bried J. Lambardi P. Luschi P. Wikelski M. Bonadonna F. (2013). Oceanic navigation in Cory’s shearwaters: evidence for a crucial role of olfactory cues for homing after displacement. *J. Exp. Biol.* 216 2798–2805. 10.1242/jeb.085738 23842626

[B50] Gagliardo A. Colombo S. Pollonara E. Casini G. Rossino M. G. Wikelski M. (2021). GPS-profiling of retrograde navigational impairments associated with hippocampal lesion in homing pigeons. *Behav. Brain Res.* 412:113408. 10.1016/j.bbr.2021.113408 34111471

[B51] Gahete M. D. Durán-Prado M. Luque R. M. Martínez-Fuentes A. J. Quintero A. Gutiérrez-Pascual E. (2009). Understanding the multifactorial control of growth hormone release by somatotropes: lessons from comparative endocrinology. *Ann. N.Y. Acad. Sci.* 1163 137–153. 10.1111/j.1749-6632.2008.03660.x 19456335

[B52] Ganea D. Delgado M. (2002). Vasoactive intestinal peptide (VIP) and pituitary adenylate cyclase-activating polypeptide (PACAP) as modulators of both innate and adaptive immunity. *Crit. Rev. Oral. Biol. Med.* 13 229–237. 10.1177/154411130201300303 12090463

[B53] Gastelum C. Perez L. Hernandez J. Le N. Vahrson I. Sayers S. (2021). Adaptive changes in the central control of energy homeostasis occur in response to variations in energy status. *Int. J. Mol. Sci.* 22:2728. 10.3390/ijms22052728 33800452 PMC7962960

[B54] Gilmartin M. R. Ferrara N. C. (2021). Pituitary adenylate cyclase-activating polypeptide in learning and memory. *Front. Cell Neurosci.* 15:663418. 10.3389/fncel.2021.663418 PMC8258392 34239418

[B55] Guerreiro L. C. F. Henrique E. P. da Silva Rosa J. B. Pereira P. D. C. de Abreu C. C. Fernandes T. N. (2021). Plasticity in the hippocampal formation of shorebirds during the wintering period: stereological analysis of parvalbumin neurons in actitis macularius. *Learn. Behav.* [Epub ahead of print], 10.3758/s13420-021-00473-6 34244975

[B56] Guigueno M. F. MacDougall-Shackleton S. A. Sherry D. F. (2016). Sex and seasonal differences in hippocampal volume and neurogenesis in brood-parasitic brown-headed cowbirds (molothrus ater). *Dev. Neurobiol.* 76 1275–1290. 10.1002/dneu.22421 27455512

[B57] Hammack S. E. Roman C. W. Lezak K. R. Kocho-Shellenberg M. Grimmig B. Falls W. A. (2010). Roles for pituitary adenylate cyclase-activating peptide (PACAP) expression and signaling in the bed nucleus of the stria terminalis (BNST) in mediating the behavioral consequences of chronic stress. *J. Mol. Neurosci.* 42 327–340. 10.1007/s12031-010-9364-7 20405238 PMC2955825

[B58] Hansson E. Westerlund A. Björklund U. Rönnbäck L. (2009). PACAP attenuates 5-HT, histamine, and ATP-evoked Ca2+ transients in astrocytes. *Neuroreport* 20 957–962. 10.1097/WNR.0b013e32832ca201 19474768

[B59] Haraguchi S. Kamata M. Tokita T. Tashiro K. I. Sato M. Nozaki M. (2019). Light-at-night exposure affects brain development through pineal allopregnanolone-dependent mechanisms. *Elife* 8:e45306. 10.7554/eLife.45306 31566568 PMC6850767

[B60] Hardy O. J. Charbonnel N. Fréville H. Heuertz M. (2003). Microsatellite allele sizes: a simple test to assess their significance on genetic differentiation. *Genetics* 163 1467–1482. 10.1093/genetics/163.4.1467 12702690 PMC1462522

[B61] Henrique E. P. de Oliveira M. A. Paulo D. C. Pereira P. D. C. Dias C. de Siqueira L. S. (2020). Contrasting migratory journeys and changes in hippocampal astrocyte morphology in shorebirds. *Eur. J. Neurosci.* 54 5687–5704. 10.1111/ejn.14781 32406131

[B62] Hicklin P. Gratto-Trevor C. L. (2020). “Semipalmated sandpiper (Calidris pusilla) version 1.0,” in *Birds of the World*, ed. Poole A. F. (Ithaca, NY: Cornell Lab of Ornithology). 10.2173/bow.semsan.01

[B63] Hwang S. N. Lee J. S. Seo K. Lee H. (2021). Astrocytic regulation of neural circuits underlying behaviors. *Cells* 10:296. 10.3390/cells10020296 33535587 PMC7912785

[B64] Iwasa T. Matsuzaki T. Tungalagsuvd A. Munkhzaya M. Yiliyasi M. Kato T. (2016). Developmental changes in the hypothalamic mRNA expression levels of PACAP and its receptor PAC1 and their sensitivity to fasting in male and female rats. *Int. J. Dev. Neurosci.* 52 33–37. 10.1016/j.ijdevneu.2016.05.003 27181029

[B65] Johnson G. C. Parsons R. May V. Hammack S. E. (2020). The role of pituitary adenylate cyclase-activating polypeptide (PACAP) signaling in the hippocampal dentate gyrus. *Front. Cell Neurosci.* 14:111. 10.3389/fncel.2020.00111 32425759 PMC7203336

[B66] Kambe Y. Yamauchi Y. Thanh Nguyen T. Thi Nguyen T. Ago Y. Shintani N. (2021). The pivotal role of pituitary adenylate cyclase-activating polypeptide for lactate production and secretion in astrocytes during fear memory. *Pharmacol. Rep.* 73 1109–1121. 10.1007/s43440-021-00222-6 33835466

[B67] Karpiesiuk A. Palus K. (2021). Pituitary adenylate cyclase-activating polypeptide (PACAP) in physiological and pathological processes within the gastrointestinal tract: a review. *Int. J. Mol. Sci.* 22:8682. 10.3390/ijms22168682 34445388 PMC8395522

[B68] Kataoka S. Takuma K. Hara Y. Maeda Y. Ago Y. Matsuda T. (2013). Autism-like behaviours with transient histone hyperacetylation in mice treated prenatally with valproic acid. *Int. J. Neuropsychopharmacol.* 16 91–103. 10.1017/S1461145711001714 22093185

[B69] Kinhult J. Andersson J. A. Uddman R. Stjärne P. Cardell L. O. (2000). Pituitary adenylate cyclase-activating peptide 38 a potent endogenously produced dilator of human airways. *Eur. Respir J.* 15 243–247. 10.1034/j.1399-3003.2000.15b04.x 10706486

[B70] Kirry A. J. Herbst M. R. Poirier S. E. Maskeri M. M. Rothwell A. C. Twining R. C. (2018). Pituitary adenylate cyclase-activating polypeptide (PACAP) signaling in the prefrontal cortex modulates cued fear learning, but not spatial working memory, in female rats. *Neuropharmacology* 133 145–154. 10.1016/j.neuropharm.2018.01.010 29353055

[B71] Kong L. Albano R. Madayag A. Raddatz N. Mantsch J. R. Choi S. (2016). Pituitary adenylate cyclase-activating polypeptide orchestrates neuronal regulation of the astrocytic glutamate-releasing mechanism system xc (.). *J. Neurochem.* 137 384–393. 10.1111/jnc.13566 26851652 PMC4878137

[B72] Levene H. (1960). *Contributionsto Probability and Statistics.* Redwood City, CA: Stanford University Press, 278–292.

[B73] Lindén A. Cardell L. O. Yoshihara S. Nadel J. A. (1999). Bronchodilation by pituitary adenylate cyclase-activating peptide and related peptides. *Eur. Respir J.* 14 443–451. 10.1034/j.1399-3003.1999.14b34.x 10515428

[B74] Magalhaes N. G. D. Diniz C. G. Diniz D. G. Henrique E. P. Pereira P. D. C. I Moraes A. M. (2017). Hippocampal neurogenesis and volume in migrating and wintering semipalmated sandpipers (*Calidris pusilla*). *PLos One* 12:e0179134. 10.1371/journal.pone.0179134 28591201 PMC5462419

[B75] Magistretti P. J. Cardinaux J. R. Martin J. L. (1998). VIP and PACAP in the CNS: regulators of glial energy metabolism and modulators of glutamatergic signaling. *Ann. N.Y. Acad. Sci.* 865 213–225. 10.1111/j.1749-6632.1998.tb11181.x 9928015

[B76] Mann R. P. Armstrong C. Meade J. Freeman R. Biro D. Guilford T. (2014). Landscape complexity influences route-memory formation in navigating pigeons. *Biol. Lett.* 10:20130885. 10.1098/rsbl.2013.0885 24451267 PMC3917332

[B77] Masmoudi-Kouki O. Gandolfo P. Castel H. Leprince J. Fournier A. Dejda A. (2007). Role of PACAP and VIP in astroglial functions. *Peptides* 28 1753–1760. 10.1016/j.peptides.2007.05.015 17655978

[B78] Mayo O. (2008). A century of hardy-weinberg equilibrium. *Twin Res. Hum. Genet.* 11 249–256. 10.1375/twin.11.3.249 18498203

[B79] McArdle B. Anderson M. (2001). Fitting multivariate models to community data: a comment on distance−based redundancy analysis. *Ecology* 82 290–297. 10.1890/0012-9658(2001)082[0290:fmmtcd]2.0.co;2

[B80] Meirmans P. G. (2020). genodive version 3.0: easy-to-use software for the analysis of genetic data of diploids and polyploids. *Mol. Ecol. Resour.* 20 1126–1131. 10.1111/1755-0998.13145 32061017 PMC7496249

[B81] Meirmans P. G. Liu S. van Tienderen P. H. (2018). The analysis of polyploid genetic data. *J. Hered* 109 283–296. 10.1093/jhered/esy006 29385510

[B82] Mendes de Lima C. P. Douglas Corrêa Pereira P. Pereira Henrique E. Augusto de Oliveira M. Carvalho Paulo D. Silva de Siqueira L. (2019). Differential change in hippocampal radial astrocytes and neurogenesis in shorebirds with contrasting migratory routes. *Front. Neuroanat* 13:82. 10.3389/fnana.2019.00082 31680881 PMC6798042

[B83] Mettler R. Segelbacher G. Schaefer H. M. (2015). Interactions between a candidate gene for migration (ADCYAP1), morphology and sex predict spring arrival in blackcap populations. *PLoS One* 10:e0144587. 10.1371/journal.pone.0144587 26684459 PMC4684316

[B84] Michel S. Itri J. Han J. H. Gniotczynski K. Colwell C. S. (2006). Regulation of glutamatergic signalling by PACAP in the mammalian suprachiasmatic nucleus. *BMC Neurosci.* 7:15. 10.1186/1471-2202-7-15 16483357 PMC1388226

[B85] Montero M. Yon L. Kikuyama S. Dufour S. Vaudry H. (2000). Molecular evolution of the growth hormone-releasing hormone/pituitary adenylate cyclase-activating polypeptide gene family. functional implication in the regulation of growth hormone secretion. *J. Mol. Endocrinol.* 25 157–168. 10.1677/jme.0.0250157 11013344

[B86] Moura R. F. Dawson D. A. Nogueira D. M. (2017). The use of microsatellite markers in neotropical studies of wild birds: a literature review. *An. Acad. Bras Cienc* 89 145–154. 10.1590/0001-3765201620160378 28177053

[B87] Mouritsen H. Heyers D. Güntürkün O. (2016). The neural basis of long-distance navigation in birds. *Annu Rev. Physiol.* 78 133–154. 10.1146/annurev-physiol-021115-105054 26527184

[B88] Mueller J. C. Pulido F. Kempenaers B. (2011). Identification of a gene associated with avian migratory behaviour. *Proc. Biol. Sci.* 278 2848–2856. 10.1098/rspb.2010.2567 21325325 PMC3145181

[B89] Murat C. B. García-Cáceres C. (2021). Astrocyte gliotransmission in the regulation of systemic metabolism. *Metabolites* 11:732. 10.3390/metabo11110732 34822390 PMC8623475

[B90] Nagy A. D. Csernus V. J. (2007). Cry1 expression in the chicken pineal gland: effects of changes in the light/dark conditions. *Gen Comp. Endocrinol.* 152 144–147. 10.1016/j.ygcen.2007.01.019 17324421

[B91] Nakahara K. Abe Y. Murakami T. Shiota K. Murakami N. (2002). Pituitary adenylate cyclase-activating polypeptide (PACAP) is involved in melatonin release via the specific receptor PACAP-r1, but not in the circadian oscillator, in chick pineal cells. *Brain Res.* 939 19–25. 10.1016/s0006-8993(02)02538-6 12020847

[B92] Nakamachi T. Farkas J. Watanabe J. Ohtaki H. Dohi K. Arata S. (2011). Role of PACAP in neural stem/progenitor cell and astrocyte–from neural development to neural repair. *Curr. Pharm Des.* 17 973–984. 10.2174/138161211795589346 21524256

[B93] Nakata M. Zhang B. Yang Y. Okada T. Shintani N. Hashimoto H. (2016). High-fat diet augments VPAC1 receptor-mediated PACAP action on the liver, inducing LAR expression and insulin resistance. *J. Diabetes Res.* 2016:9321395. 10.1155/2016/9321395 28044141 PMC5156820

[B94] Nei M. (1978). Estimation of average heterozygosity and genetic distance from a small number of individuals. *Genetics* 89 583–590. 10.1093/genetics/89.3.583 17248844 PMC1213855

[B95] Nelder J. Wedderburn R. (1972). Generalized linear models. *J. R.Stat. Soc. Ser. A* 135:370.

[B96] Nguyen T. T. Kambe Y. Kurihara T. Nakamachi T. Shintani N. Hashimoto H. (2020). Pituitary adenylate cyclase-activating polypeptide in the ventromedial hypothalamus is responsible for food intake behavior by modulating the expression of agouti-related peptide in mice. *Mol. Neurobiol.* 57 2101–2114. 10.1007/s12035-019-01864-7 31927724

[B97] Nóbrega P. F. Aguiar J. A. Figueira J. E. (2015). First records of charadrius semipalmatus, bonaparte 1825 (charadriidae) and gelochelidon nilotica gmelin 1789 (sternidae) in the state of minas gerais, brazil. *Braz J. Biol.* 75 451–454. 10.1590/1519-6984.17013 26132031

[B98] Norrholm S. D. Das M. Légrádi G. (2005). Behavioral effects of local microinfusion of pituitary adenylate cyclase activating polypeptide (PACAP) into the paraventricular nucleus of the hypothalamus (PVN). *Regul Pept.* 128 33–41. 10.1016/j.regpep.2004.12.023 15721485 PMC1950325

[B99] Nowak J. Z. Zawilska J. B. (2003). PACAP in avians: origin, occurrence, and receptors–pharmacological and functional considerations. *Curr. Pharm Des.* 9 467–481. 10.2174/1381612033391586 12570810

[B100] Olkowski A. A. Classen H. L. (1998). Safety of isoflurane anaesthesia in high risk avian patients. *Vet. Rec.* 143 82–83. 10.1136/vr.143.3.82 9717227

[B101] Perez V. Bouschet T. Fernandez C. Bockaert J. Journot L. (2005). Dynamic reorganization of the astrocyte actin cytoskeleton elicited by cAMP and PACAP: a role for phosphatidylInositol 3-kinase inhibition. *Eur. J. Neurosci.* 21 26–32. 10.1111/j.1460-9568.2004.03845.x 15654840

[B102] Peterson M. P. Abolins-Abols M. Atwell J. W. Rice R. J. Milá B. Ketterson E. D. (2013). Variation in candidate genes CLOCK and ADCYAP1 does not consistently predict differences in migratory behavior in the songbird genus junco. *F1000Res* 2:115. 10.12688/f1000research.2-115.v1 24627781 PMC3907158

[B103] Piersma T. Wiersma P. (1996). “Order charadriiformes. Family charadriidae (Plovers),” in *Handbook of the Birds of the World*, Vol. 3, eds del Hoyo J. Elliot A. Sargatal J. (Spain: Hoatzin to Auks. Lynx Edicions, Barcelona), 384–443.

[B104] Pillai A. G. de Jong D. Kanatsou S. Krugers H. Knapman A. Heinzmann J. M. (2012). Dendritic morphology of hippocampal and amygdalar neurons in adolescent mice is resilient to genetic differences in stress reactivity. *PLoS One* 7:e38971. 10.1371/journal.pone.0038971 22701737 PMC3373517

[B105] Primmer C. R. Møller A. P. Ellegren H. (1996). A wide-range survey of cross-species microsatellite amplification in birds. *Mol. Ecol.* 5 365–378. 10.1111/j.1365-294x.1996.tb00327.x 8688957

[B106] Prisco M. Rosati L. Agnese M. Aceto S. Andreuccetti P. Valiante S. (2019). Pituitary adenylate cyclase-activating polypeptide in the testis of the quail coturnix coturnix: expression, localization, and phylogenetic analysis. *Evol. Dev.* 21 145–156. 10.1111/ede.12285 30791203

[B107] Ralston J. Lorenc L. Montes M. DeLuca W. V. Kirchman J. J. Woodworth B. K. (2019). Length polymorphisms at two candidate genes explain variation of migratory behaviors in blackpoll warblers. *Ecol. Evol.* 9 8840–8855. 10.1002/ece3.5436 31410284 PMC6686290

[B108] Reed J. M. Oring L. W. (1993). Philopatry, site fidelity, dispersal, and survival of spotted sandpipers. *AUK* 110 541–551. 10.2307/4088418

[B109] Reed J. Oring L. Gray E. (2013). “Spotted sandpiper (actitis macularius), version 2.0,” in *The Birds of North America. In The Birds of North America*, ed. Poole A. (Ithaca, NY: Cornell Lab of Ornithology).

[B110] Rensel M. A. Ellis J. M. Harvey B. Schlinger B. A. (2015). Sex, estradiol, and spatial memory in a food-caching corvid. *Horm Behav.* 75 45–54. 10.1016/j.yhbeh.2015.07.022 26232613 PMC4648678

[B111] Ribeiro J. B. M. (2001). *Micrometeorologia do manguezal e o impacto do desmatamento em Bragança-PA [tese]*. São Carlos: Escola de Engenharia de São Carlos; Universidade de São Paulo (USP). 10.11606/T.18.2001.tde-11112015-122408

[B112] Riley D. E. Krieger J. N. (2009). UTR dinucleotide simple sequence repeat evolution exhibits recurring patterns including regulatory sequence motif replacements. *Gene* 429 80–86. 10.1016/j.gene.2008.09.030 18955121 PMC2633293

[B113] Riou S. Chastel O. Lacroix A. Hamer K. C. (2010). Stress and parental care: prolactin responses to acute stress throughout the breeding cycle in a long-lived bird. *Gen Comp. Endocrinol.* 168 8–13. 10.1016/j.ygcen.2010.03.011 20331990

[B114] Rodrigues A. A. F. (2000). Seasonal abundance of neartic shorebirds in the gulf of maranhão, brazil. *J. Field Ornithol.* 71 665–675. 10.1648/0273-8570-71.4.665

[B115] Rodrigues A. A. F. (2006). *Aves da Reserva Biológica do Lago Piratuba e Entorno, Amapá, Brasil. Inventário Biológico das Áreas do Sucuriju e Região do lagos, Amapá: Relatório Final PROBIO.* Macapá: Instituto de Pesquisas Científicas e Tecnológicas do Estado do Amapá, 188–195.

[B116] Rudecki A. P. Gray S. L. (2016). PACAP in the defense of energy homeostasis. *Trends Endocrinol. Metab.* 27 620–632. 10.1016/j.tem.2016.04.008 27166671

[B117] Sadanandan N. Cozene B. Park Y. J. Farooq J. Kingsbury C. Wang Z. J. (2021). Pituitary adenylate cyclase-activating polypeptide: a potent therapeutic agent in oxidative stress. *Antioxidants (Basel)* 10:354. 10.3390/antiox10030354 33653014 PMC7996859

[B118] Saino N. Bazzi G. Gatti E. Caprioli M. Cecere J. G. Possenti C. D. (2015). Polymorphism at the clock gene predicts phenology of long-distance migration in birds. *Mol. Ecol.* 24 1758–1773. 10.1111/mec.13159 25780812

[B119] Salkind N. (2007). *Encyclopedia of Measurement and Statistics.* California: Sage Publications, Inc.

[B120] Samojłowicz D. Twarowska-Małczyńska J. Borowska-Solonynko A. Poniatowski Ł Sharma N. Olczak M. (2019). Presence of *Toxoplasma gondii* infection in brain as a potential cause of risky behavior: a report of 102 autopsy cases. *Eur. J. Clin. Microbiol. Infect. Dis.* 38 305–317. 10.1007/s10096-018-3427-z 30470966 PMC6514116

[B121] Saper C. B. Sawchenko P. E. (2003). Magic peptides, magic antibodies: guidelines for appropriate controls for immunohistochemistry. *J. Comp. Neurol.* 465 161–163. 10.1002/cne.10858 12949777

[B122] Schmitt O. Preusse S. Haas S. J. (2004). Comparison of contrast, sensitivity and efficiency of signal amplified and nonamplified immunohistochemical reactions suitable for videomicroscopy-based quantification and neuroimaging. *Brain Res. Protoc.* 12 157–171. 10.1016/j.brainresprot.2003.10.003 15013467

[B123] Schubert M. L. (2003). Gastric secretion. *Curr. Opin. Gastroenterol.* 19 519–525. 15703599 10.1097/00001574-200311000-00002

[B124] Schuelke M. (2000). An economic method for the fluorescent labeling of PCR fragments. *Nat. Biotechnol.* 18 233–234. 10.1038/72708 10657137

[B125] Schweitzer L. Renehan W. E. (1997). The use of cluster analysis for cell typing. *Brain Res. Brain Res. Protoc.* 1 100–108. 9385054 10.1016/s1385-299x(96)00014-1

[B126] Seo H. Lee K. (2016). Epac2 contributes to PACAP-induced astrocytic differentiation through calcium ion influx in neural precursor cells. *BMB Rep.* 49 128–133. 10.5483/bmbrep.2016.49.2.202 26645637 PMC4915117

[B127] Serrano I. (2010). *Distribuição e Conservação de Aves Migratórias Neárticas da Ordem Charadriiformes (Famílias Charadriidae e Scolopacidae) No Brasil.* Brazil: department of zoology. universidade federal do pará museu paraense emílio goeldi programa de pós-graduação em zoologia curso de doutorado em zoologia, belém (PA), 174.

[B128] Shao S. Yang Y. Yuan G. Zhang M. Yu X. (2013). Signaling molecules involved in lipid-induced pancreatic beta-cell dysfunction. *DNA Cell Biol.* 32 41–49. 10.1089/dna.2012.1874 23347443 PMC3557433

[B129] Shapiro S. Wilk M. (1965). An analysis of variance test for normality (complete samples). *Biometrika* 52:591.

[B130] Sharma A. Singh D. Das S. Kumar V. (2018a). Hypothalamic and liver transcriptome from two crucial life-history stages in a migratory songbird. *Exp. Physiol.* 103 559–569. 10.1113/EP086831 29380464

[B131] Sharma A. Singh D. Malik S. Gupta N. J. Rani S. Kumar V. (2018b). Difference in control between spring and autumn migration in birds: insight from seasonal changes in hypothalamic gene expression in captive buntings. *Proc. Biol. Sci.* 285:20181531. 10.1098/rspb.2018.1531 30158302 PMC6125905

[B132] Shu S. Ju G. Fan L. (1988). The glucose oxidase-DAB-nickel method in peroxidase histochemistry of the nervous system. *Neurosci. Lett.* 85 169–171. 10.1016/0304-3940(88)90346-1 3374833

[B133] Simon Á Oláh J. Komlósi I. Jávor A. Németh J. Szilvássy Z. (2017). Changes in expression of neuropeptides and their receptors in the hypothalamus and gastrointestinal tract of calorie restricted hens. *Acta Biol. Hung* 68 237–247. 10.1556/018.68.2017.3.1 28901800

[B134] Skagen S. Sharpe P. Waltermire R. Dillon M. (1999). *Biogeographical Profiles of Shorebird Migration in Midcontinental North America*. Springfield, VA: U.S. Dept. of the Interior, U.S. Geological Survey, 178.

[B135] Song S. Dey D. K. Holsinger K. E. (2011). Genetic diversity of microsatellite loci in hierarchically structured populations. *Theor. Popul. Biol.* 80 29–37. 10.1016/j.tpb.2011.04.004 21575649 PMC3124608

[B136] Steinmeyer C. Mueller J. C. Kempenaers B. (2009). Search for informative polymorphisms in candidate genes: clock genes and circadian behaviour in blue tits. *Genetica* 136 109–117. 10.1007/s10709-008-9318-y 18792794 PMC2832883

[B137] Surbhi Rastogi A. Malik S. Rani S. Kumar V. (2016). Changes in brain peptides associated with reproduction and energy homeostasis in photosensitive and photorefractory migratory redheaded buntings. *Gen Comp. Endocrinol.* 23 67–75. 10.1016/j.ygcen.2016.03.031 27038875

[B138] Tachibana T. Sugimoto I. Ogino M. Khan M. S. Masuda K. Ukena K. (2015). Central administration of chicken growth hormone-releasing hormone decreases food intake in chicks. *Physiol. Behav.* 139 195–201. 10.1016/j.physbeh.2014.11.043 25449398

[B139] Thalheimer W. Cook S. (2002). How to calculate effect sizes from published research: a simplified methodology. *Work Res.* 1 1–9.

[B140] Toth D. Szabo E. Tamas A. Juhasz T. Horvath G. Fabian E. (2020). Protective effects of PACAP in peripheral organs. *Front. Endocrinol. (Lausanne)* 11:377. 10.3389/fendo.2020.00377 32765418 PMC7381171

[B141] van Toor M. L. Hedenström A. Waldenström J. Fiedler W. Holland R. A. Thorup K. (2013). Flexibility of continental navigation and migration in European mallards. *PLoS One* 8:e72629. 10.1371/journal.pone.0072629 24023629 PMC3758317

[B142] Vaudry D. Falluel-Morel A. Bourgault S. Basille M. Burel D. Wurtz O. (2009). Pituitary adenylate cyclase-activating polypeptide and its receptors: 20 years after the discovery. *Pharmacol. Rev.* 61 283–357. 10.1124/pr.109.001370 19805477

[B143] Vélez E. J. Unniappan S. (2020). A comparative update on the neuroendocrine regulation of growth hormone in vertebrates. *Front. Endocrinol. (Lausanne)* 11:614981. 10.3389/fendo.2020.614981 33708174 PMC7940767

[B144] Ward J. (1963). Hierarchical grouping to optimize an objective function. *J. Am. Stat. Assoc.* 58 236–244.

[B145] West M. J. (2002). Design-based stereological methods for counting neurons. *Prog. Brain Res.* 135 43–51. 10.1016/S0079-6123(02)35006-4 12143362

[B146] Williams T. Williams W. (1978). An oceanic mass migration of land birds. *Sci. Am.* 239 173–176. 10.1038/scientificamerican1078-166

[B147] Wiltschko R. Wiltschko W. (2012). Magnetoreception. *Adv. Exp. Med. Biol.* 739 126–141. 22399399 10.1007/978-1-4614-1704-0_8

[B148] Wiltschko R. Wiltschko W. (2019). Magnetoreception in birds. *J. R. Soc. Interface* 16:20190295. 10.1098/rsif.2019.0295 PMC6769297 31480921

[B149] Wiltschko W. Wiltschko R. (2012). Global navigation in migratory birds: tracks, strategies, and interactions between mechanisms. *Curr. Opin. Neurobiol.* 22 328–335. 10.1016/j.conb.2011.12.012 22244742

[B150] Winters S. J. Moore J. P. (2020). PACAP: a regulator of mammalian reproductive function. *Mol. Cell. Endocrinol.* 518:110912. 10.1016/j.mce.2020.110912 32561449 PMC7606562

[B151] Yamada J. Jinno S. (2013). Novel objective classification of reactive microglia following hypoglossal axotomy using hierarchical cluster analysis. *J. Comp. Neurol.* 521 1184–1201. 10.1002/cne.23228 22987820

