# Supplementary material for: Shorebirds’ Longer Migratory Distances Are Associated With Larger ADCYAP1 Microsatellites and Greater Morphological Complexity of Hippocampal Astrocytes
Source: Front Psychol. 2022 Feb 4;12:784372. doi: 10.3389/fpsyg.2021.784372 (PMC8855117; doi:10.3389/fpsyg.2021.784372)
Supplement: Supplementary file 1 [file Table_1.DOCX]

Table S1: Morphometric parameters of three-dimensional reconstructed astrocytes.

| Branched Structure Analysis | |
| --- | --- |
| Segment | Any portion of microglial branched structure with endings that are either nodes or terminations with no intermediate nodes. |
| Segments/mm | Number of segments/total length of the segments expressed in millimeters. |
| No of trees | Number of trees in the astrocyte |
| Total No of segments | Refer to the total number of segments in the tree. |
| Branch length | Total length of the line segments used to trace the branch of interest. |
| Total branch length | Total length for all branches in the tree.  Mean = [Length] / [Number of branches] |
| Tortuosity | = [Actual length of the segment]/[Distance between the endpoints of the segment]. The smallest value is 1; this represents a straight segment. Tortuosity allows segments of different lengths to be compared in terms of the complexity of the paths they take. |
| Surface Area | Computed by modeling each branch as a frustum (truncated right circular cone). |
| Branch volume | Computed by modeling each piece of each branch as a frustum. |
| Total branch volume | Total volume for all branches in the tree. |
| Base Diameter of Primary Branch | Diameter at the start of the 1^st^ segment. |
| Planar Angle | Computed based on the endpoints of the segments. It refers to the change in direction of a segment relative to the previous segment. |
| Fractal dimension | the “k-dim” of the fractal analysis, describes how the structure of interest fills space. Significant statistical differences in K-dim suggest morphological dissimilarities. |
| Complexity | Complexity = [Sum of the terminal orders + Number of terminals] × [Total branch length / Number of primary branches] |
| Convex hull | Convex hull measures the size of the branching field by interpreting a branched structure as a solid object controlling a given amount of physical space. The amount of physical space is defined in terms of convex-hull volume, surface area, area, and or perimeter. |
| Vertex Va, Vb, Vc | Describes the overall structure of a branched object based on topological and metrical properties. Root (or origin) point: For neurons, microglia or astrocytes, the origin is the point at which the structure is attached to the soma. Main types of vertices: V_d_ (bifurcation) or V_t_ (trifurcation): Nodal (or branching) points. V_p_: Terminal (or pendant) vertices. V_a_: primary vertices connecting 2 pendant vertices; V_b_: secondary vertices connecting 1 pendant vertex (V_p_) to 1 bifurcation (V_d_) or 1 trifurcation (V_t_); V_c_: tertiary vertices connecting either 2 bifurcations (V_d_), 2 trifurcations (V_t_), or 1 bifurcation (V_d_) and 1 trifurcation (V_t_). In the present report we measure the number of vertices Va, Vb and Vc. |
