# Supplementary material for: Shorebirds’ Longer Migratory Distances Are Associated With Larger ADCYAP1 Microsatellites and Greater Morphological Complexity of Hippocampal Astrocytes
Source: Front Psychol. 2022 Feb 4;12:784372. doi: 10.3389/fpsyg.2021.784372 (PMC8855117; doi:10.3389/fpsyg.2021.784372)
Supplement: Supplementary file 2 [file Table_2.DOCX]

**S2 Table:** Shapiro-Wilk Normality Test (p> 0.05) for stereological, morphometric and microsatellite variables for the study of migratory and non-migratory birds.

|  |  | Levene's Error Variation Equality Test | | | Shapiro-Wilk Normality Tests | | |
| --- | --- | --- | --- | --- | --- | --- | --- |
|  | F | Df1 | df2 | Sig. | Stat | df | Sig. |
| Complexity | 79.430 | 7 | 1069 | **.000** | .676 | 102 | **.000** |
| Micros | 1.238 | 3 | 98 | .300 | .924 | 102 | **.000** |
| LogComplexity | 9.428 | 7 | 1069 | **.000** | .988 | 102 | .507 |
| LogMicros | - | - | - | - | .926 | 102 | **.000** |
