# Supplementary material for: Shorebirds’ Longer Migratory Distances Are Associated With Larger ADCYAP1 Microsatellites and Greater Morphological Complexity of Hippocampal Astrocytes
Source: Front Psychol. 2022 Feb 4;12:784372. doi: 10.3389/fpsyg.2021.784372 (PMC8855117; doi:10.3389/fpsyg.2021.784372)
Supplement: Supplementary file 3 [file Table_3.DOCX]

**S3 Table**: Size of microsatellite alleles for the *ADCYAP1* gene found in four species (*C. collaris*, *C semipalmatus*, *C. pusilla* and *A macularius*) with different migratory behaviors.

| Species | Allele Size of ADCYAP1 | | Specie | Allele Size of ADCYAP1 | | Specie | Allele Size of ADCYAP1 | | Specie | Allele Size of ADCYAP1 | |
| --- | --- | --- | --- | --- | --- | --- | --- | --- | --- | --- | --- |
|  | Allele 1 | Allele 2 |  | Allele 1 | Allele 2 |  | Allele 1 | Allele 2 |  | Allele 1 | Allele 2 |
| *A. macularia 01* | 196 | 196 | *C. pusilla 01* | 182 | 188 | *C. semipalmatus 01* | 178 | 182 | *C. collaris 01* | 172 | 174 |
| *A. macularia 02* | 196 | 196 | *C. pusilla 02* | 186 | 190 | *C. semipalmatus 02* | 174 | 176 | *C. collaris 02* | 172 | 174 |
| *A. macularia 03* | 194 | 194 | *C. pusilla 03* | 188 | 188 | *C. semipalmatus 03* | 176 | 178 | *C. collaris 03* | 172 | 172 |
| *A. macularia 04* | 194 | 198 | *C. pusilla 04* | 184 | 188 | *C. semipalmatus 04* | 174 | 178 | *C. collaris 04* | 182 | 182 |
| *A. macularia 05* | 196 | 196 | *C. pusilla 05* | 182 | 186 | *C. semipalmatus 05* | 174 | 178 | *C. collaris 05* | 172 | 174 |
| *A. macularia 06* | 198 | 198 | *C. pusilla 06* | 184 | 192 | *C. semipalmatus 06* | 174 | 178 | *C. collaris 06* | 172 | 172 |
| *A. macularia 07* | 194 | 194 | *C. pusilla 07* | 184 | 188 | *C. semipalmatus 07* | 176 | 176 | *C. collaris 07* | 172 | 174 |
| *A. macularia 08* | 196 | 204 | *C. pusilla 08* | 188 | 188 | *C. semipalmatus 08* | 176 | 176 | *C. collaris 08* | 170 | 172 |
| *A. macularia 09* | 192 | 198 | *C. pusilla 09* | 186 | 188 | *C. semipalmatus 09* | 176 | 176 | *C. collaris 09* | 168 | 172 |
| *A. macularia 10* | 194 | 196 | *C. pusilla 10* | 186 | 186 | *C. semipalmatus 10* | 178 | 178 | *C. collaris 10* | 170 | 172 |
| *A. macularia 11* | 192 | 192 | *Calidris pusilla 11* | 186 | 188 | *C. semipalmatus 11* | 174 | 178 | *C. collaris 11* | 172 | 172 |
| *A. macularia 12* | 194 | 198 | *C. pusilla 12* | 186 | 188 | *C. semipalmatus 12* | 178 | 178 | *C. collaris 12* | 170 | 174 |
| - | - | - | *C. pusilla 13* | 182 | 188 | *C. semipalmatus 13* | 196 | 196 | *C. collaris 13* | 174 | 174 |
| - | - | - | *C. pusilla 14* | 184 | 186 | - | - | - | *C. collaris 14* | 174 | 176 |
