# Supplementary material for: Shorebirds’ Longer Migratory Distances Are Associated With Larger ADCYAP1 Microsatellites and Greater Morphological Complexity of Hippocampal Astrocytes
Source: Front Psychol. 2022 Feb 4;12:784372. doi: 10.3389/fpsyg.2021.784372 (PMC8855117; doi:10.3389/fpsyg.2021.784372)
Supplement: Supplementary file 4 [file Table_4.DOCX]

**S4 Table:** Genetic diversity of ADCYAP1 locus across the four species populations analyzed.

| Species | N | A | Alele Range | N° of Exclusive Alleles | *Ho* | *He* | HW eq | N° of Homozygotes |
| --- | --- | --- | --- | --- | --- | --- | --- | --- |
| *A. macularius** | 12 | 5 | 192-204 | 3 | 0.417 | 0.792 | *p*=0.0208 | 7 (58%) |
| *C. pusilla* | 14 | 6 | 182-192 | 4 | 0.786 | 0.755 | *p*=0.9436 | 3 (21%) |
| *C. semipalmatus** | 13 | 5 | 174-182 | 1 | 0.538 | 0.750 | *p*=0.0001 | 6 (46%) |
| *C. colaris* | 14 | 6 | 168-182 | 3 | 0.643 | 0.712 | *p*=0.4190 | 5 (35%) |

N, samples per population; A, number of alleles; *H_O_*, observed heterozygosity; *H_E_*, expected heterozygosity; HW eq, significance values of deviations from Hardy-Weinberg equilibrium. * Significant deviations from Hardy-Weinberg equilibrium.
