# Supplementary material for: Shorebirds’ Longer Migratory Distances Are Associated With Larger ADCYAP1 Microsatellites and Greater Morphological Complexity of Hippocampal Astrocytes
Source: Front Psychol. 2022 Feb 4;12:784372. doi: 10.3389/fpsyg.2021.784372 (PMC8855117; doi:10.3389/fpsyg.2021.784372)
Supplement: Supplementary file 5 [file Table_5.DOCX]

**S5 Table:** FST (below diagonal) and RST (above diagonal) values for population differentiation at ADCYAP1 locus.

|  | *A. macularius* | *C. pusilla* | *C. semipalmatus* | *C. colaris* |
| --- | --- | --- | --- | --- |
| *A. macularius* | --------------- | 0.8717* | 0.8870* | 0.9694* |
| *C. pusilla* | 0.2307* | --------------- | 0.6523* | 0.9233* |
| *C. semipalmatus* | 0.2217* | 0.2477* | --------------- | 0.3834* |
| *C. colaris* | 0.2586* | 0.2615* | 0.2214* | --------------- |

**p*<0.01
