# Supplementary material for: Shorebirds’ Longer Migratory Distances Are Associated With Larger ADCYAP1 Microsatellites and Greater Morphological Complexity of Hippocampal Astrocytes
Source: Front Psychol. 2022 Feb 4;12:784372. doi: 10.3389/fpsyg.2021.784372 (PMC8855117; doi:10.3389/fpsyg.2021.784372)
Supplement: Supplementary file 6 [file Table_6.DOCX]

**S6 Table:** Association of the migratory distance of *C. collaris,* *C. semipalmatus, C. pusilla and A. macularius* with the size of the ADCYAP1 gene microsatellites.

|  | | | Microsatellites | Migratory Distance |
| --- | --- | --- | --- | --- |
| Sperman | Microsatellites | Coefficient correlations | 1.000 | .915^**^ |
|  |  | Sig. (2 extremidades) | . | .000 |
|  |  | N | 102 | 102 |
|  | Migratory Distance | Correlações de coeficiente | .915^**^ | 1.000 |
|  |  | Sig. (2 extremidades) | .000 | . |
|  |  | N | 102 | 102 |
| **. Indicates that the correlation is significant at the 0.01 level (2 ends). | | | | |
