# Supplementary material for: Shorebirds’ Longer Migratory Distances Are Associated With Larger ADCYAP1 Microsatellites and Greater Morphological Complexity of Hippocampal Astrocytes
Source: Front Psychol. 2022 Feb 4;12:784372. doi: 10.3389/fpsyg.2021.784372 (PMC8855117; doi:10.3389/fpsyg.2021.784372)
Supplement: Supplementary file 7 [file Table_7.DOCX]

**S7 Table:** Analysis of Variance of Multivariate Permutation of Microsatellites of *C. collaris*, *C. semipalmatus*, *C. pusilla* and *A. macularius*.

| Search | df | SS | MS | Pseudo F | p | Component | Var | SD |
| --- | --- | --- | --- | --- | --- | --- | --- | --- |
| Es | 3 | 7.26E+03 | 2.42E+03 | 180.98 | 0.0001 | 9001 | 9.45E+01 | 9.7191 |
| Res | 98 | 1.31E+03 | 1.34E+01 |  |  |  | 1.34E+01 | 3.6558 |
| Total | 101 | 8.57E+03 |  |  |  |  |  |  |
| Pairwise comparisons | | | | | | | | |
| Groups |  |  | t | P(perm) | Unique perms | |  |  |
| *C. collaris, C. semipalmatus* | | | 4.0314 | 0.0001 | 57 |  |  |  |
| *C. collaris, C. pusilla* | |  | 18.392 | 0.0001 | 50 |  |  |  |
| *C. collaris, A. Macularius* | |  | 27.523 | 0.0001 | 137 |  |  |  |
| *C. semipalmatus, C. pusilla* | | | 7.122 | 0.0001 | 76 |  |  |  |
| *C. semipalmatus, A. Macularius* | | | 13.446 | 0.0001 | 119 |  |  |  |
| *C. pusilla, A. Macularius* | |  | 12.951 | 0.0001 | 66 |  |  |  |
| Average distance between / within groups | | | | | | | | |
|  |  | *C. collaris* | *C. semipalmatus* | | *C. pusilla* | *A. Macularius* | |  |
| *C. collaris* | | 2.9402 |  | |  |  | |  |
| *C. semipalmatus* | | 5.8133 | 4.7333 | |  |  | |  |
| *C. pusilla* | | 13.481 | 9.9378 | | 2.4729 |  | |  |
| *A. Macularius* | | 22.541 | 17.673 | | 9.0596 | 2.83 | |  |

The pseudo - F statistics were calculated for each term using direct analogues for univariate mean square expectations (EMS); p values were obtained using 9999 permutations under an unrestricted model. Each term is identified as contributing a fixed component to the general model; "Var" provides the estimated sizes of the components of the variation, based on multivariate analogs for the classic ANOVA unbiased estimators; "SD" gives the square root of these values.
