# Supplementary material for: Shorebirds’ Longer Migratory Distances Are Associated With Larger ADCYAP1 Microsatellites and Greater Morphological Complexity of Hippocampal Astrocytes
Source: Front Psychol. 2022 Feb 4;12:784372. doi: 10.3389/fpsyg.2021.784372 (PMC8855117; doi:10.3389/fpsyg.2021.784372)
Supplement: Supplementary file 8 [file Table_8.DOCX]

| **S8 Table: Independent general linear model (GLM) and paired Sidak tests for the morphological complexity of the FH astrocytes of migratory and non-migratory birds.** | | | | | | | | | | | | | | | | | | | | | | | | |  |  |  |  |
| --- | --- | --- | --- | --- | --- | --- | --- | --- | --- | --- | --- | --- | --- | --- | --- | --- | --- | --- | --- | --- | --- | --- | --- | --- | --- | --- | --- | --- |
| Dependent variable: LogComplexidade | | | | | | | | | | | | | | | | | | | | | | | | |  |  |  |  |
| Source | | | | Type III Sum of Squares | | | | df | | Square Average | | | | F | | | | | | Sig. | | Square partial Eta | | |  |  |  |  |
| Corrected model | | | | 137.517^a^ | | | | 7 | | 19.645 | | | | 211.902 | | | | | | **.000** | | .581 | | |  |  |  |  |
| Interception | | | | 10617.009 | | | | 1 | | 10617.009 | | | | 114519.787 | | | | | | **.000** | | .991 | | |  |  |  |  |
| Species | | | | 45.364 | | | | 3 | | 15.121 | | | | 163.105 | | | | | | **.000** | | .314 | | |  |  |  |  |
| Types | | | | 80.582 | | | | 1 | | 80.582 | | | | 869.190 | | | | | | **.000** | | .448 | | |  |  |  |  |
| Species vs Types | | | | .493 | | | | 3 | | .164 | | | | 1.773 | | | | | | .151 | | .005 | | |  |  |  |  |
| Error | | | | 99.106 | | | | 1069 | | .093 | | | |  | | | | | |  | |  | | |  |  |  |  |
| Total | | | | 14215.775 | | | | 1077 | |  | | | |  | | | | | |  | |  | | |  |  |  |  |
| Total corrected | | | | 236.622 | | | | 1076 | |  | | | |  | | | | | |  | |  | | |  |  |  |  |
| Pairwise comparisons | | | | | | | | | | | | | | | | | | | | | | | | |  |  |  |  |
| Type | Species | | | Species | | | | | Mean difference (I-J) | | | Standard error | | | | Sig.^b^ | | | 95% confidence interval for the difference ^b^ | | | | | | | |  |  |
|  |  |  |  |  |  |  |  |  |  |  |  |  |  |  |  |  |  |  | Inferior limit | | | | | Superior limit | | |  |  |
| 1 | *C. collaris* | | | *C. semipalmatus* | | | | | -.243^*^ | | | .055 | | | | **.000** | | | -.387 | | | | | -.099 | | |  |  |
|  |  |  |  | *C. pusilla* | | | | | -.602^*^ | | | .052 | | | | **.000** | | | -.738 | | | | | -.465 | | |  |  |
|  |  |  |  | *A. macularius* | | | | | -.494^*^ | | | .042 | | | | **.000** | | | -.605 | | | | | -.384 | | |  |  |
|  | *C. semipalmatus* | | | *C. collaris* | | | | | .243^*^ | | | .055 | | | | **.000** | | | .099 | | | | | .387 | | |  |  |
|  |  |  |  | *C. pusilla* | | | | | -.359^*^ | | | .064 | | | | **.000** | | | -.527 | | | | | -.190 | | |  |  |
|  |  |  |  | *A. macularius* | | | | | -.251^*^ | | | .056 | | | | **.000** | | | -.400 | | | | | -.103 | | |  |  |
|  | *C. pusilla* | | | *C. collaris* | | | | | .602^*^ | | | .052 | | | | **.000** | | | .465 | | | | | .738 | | |  |  |
|  |  |  |  | *C. semipalmatus* | | | | | .359^*^ | | | .064 | | | | **.000** | | | .190 | | | | | .527 | | |  |  |
|  |  |  |  | *A. macularius* | | | | | .107 | | | .053 | | | | .241 | | | -.034 | | | | | .248 | | |  |  |
|  | *A. macularius* | | | *C. collaris* | | | | | .494^*^ | | | .042 | | | | **.000** | | | .384 | | | | | .605 | | |  |  |
|  |  |  |  | *C. semipalmatus* | | | | | .251^*^ | | | .056 | | | | **.000** | | | .103 | | | | | .400 | | |  |  |
|  |  |  |  | *C. pusilla* | | | | | -.107 | | | .053 | | | | .241 | | | -.248 | | | | | .034 | | |  |  |
| 2 | *C. collaris* | | | *C. semipalmatus* | | | | | -.154^*^ | | | .032 | | | | **.000** | | | -.238 | | | | | -.070 | | |  |  |
|  |  |  |  | *C. pusilla* | | | | | -.621^*^ | | | .033 | | | | **.000** | | | -.709 | | | | | -.533 | | |  |  |
|  |  |  |  | *A. macularius* | | | | | -.403^*^ | | | .035 | | | | **.000** | | | -.495 | | | | | -.311 | | |  |  |
|  | *C. semipalmatus* | | | *C. collaris* | | | | | .154^*^ | | | .032 | | | | **.000** | | | .070 | | | | | .238 | | |  |  |
|  |  |  |  | *C. pusilla* | | | | | -.467^*^ | | | .029 | | | | **.000** | | | -.543 | | | | | -.392 | | |  |  |
|  |  |  |  | *A. macularius* | | | | | -.249^*^ | | | .030 | | | | **.000** | | | -.329 | | | | | -.170 | | |  |  |
|  | *C. pusilla* | | | *C. collaris* | | | | | .621^*^ | | | .033 | | | | **.000** | | | .533 | | | | | .709 | | |  |  |
|  |  |  |  | *C. semipalmatus* | | | | | .467^*^ | | | .029 | | | | **.000** | | | .392 | | | | | .543 | | |  |  |
|  |  |  |  | *A. macularius* | | | | | .218^*^ | | | .032 | | | | **.000** | | | .134 | | | | | .302 | | |  |  |
|  | *A. macularius* | | | *C. collaris* | | | | | .403^*^ | | | .035 | | | | **.000** | | | .311 | | | | | .495 | | |  |  |
|  |  |  |  | *C. semipalmatus* | | | | | .249^*^ | | | .030 | | | | **.000** | | | .170 | | | | | .329 | | |  |  |
|  |  |  |  | *C. pusilla* | | | | | -.218^*^ | | | .032 | | | | **.000** | | | -.302 | | | | | -.134 | | |  |  |
| Species | | Types | | | | Types | | Mean difference (I-J) | | | | | Standard error | | | | Sig.^b^ | | | 95% confidence interval for the difference ^b^ | | | | | | | | |
|  |  |  |  |  |  |  |  |  |  |  |  |  |  |  |  |  |  |  |  | Inferior limit | | | | | Superior limit | | | |
| *C. collaris* | | 1 | | | | 2 | | .613^*^ | | | | | .038 | | | | **.000** | | | .539 | | | | | .688 | | | |
|  |  | 2 | | | | 1 | | -.613^*^ | | | | | .038 | | | | **.000** | | | -.688 | | | | | -.539 | | | |
| *C. semipalmatus* | | 1 | | | | 2 | | .703^*^ | | | | | .051 | | | | **.000** | | | .604 | | | | | .802 | | | |
|  |  | 2 | | | | 1 | | -.703^*^ | | | | | .051 | | | | **.000** | | | -.802 | | | | | -.604 | | | |
| *C. pusilla* | | 1 | | | | 2 | | .594^*^ | | | | | .048 | | | | **.000** | | | .499 | | | | | .689 | | | |
|  |  | 2 | | | | 1 | | -.594^*^ | | | | | .048 | | | | **.000** | | | -.689 | | | | | -.499 | | | |
| *A. macularius* | | 1 | | | | 2 | | .705^*^ | | | | | .039 | | | | **.000** | | | .628 | | | | | .781 | | | |
|  |  | 2 | | | | 1 | | -.705^*^ | | | | | .039 | | | | **.000** | | | -.781 | | | | | -.628 | | | |
| Species | | | Species | | | | Mean difference (I-J) | | | Standard error | | | | Sig.^b^ | | | | 95% confidence interval for the difference^b^ | | | | | | | | | |  |
|  |  |  |  |  |  |  |  |  |  |  |  |  |  |  |  |  |  | Inferior limit | | | | Superior limit | | | | | |  |
| *C. collaris* | | | *C. semipalmatus* | | | | -.198^*^ | | | .032 | | | | **.000** | | | | -.282 | | | | -.115 | | | | | |  |
|  |  |  | *C. pusilla* | | | | -.611^*^ | | | .031 | | | | **.000** | | | | -.692 | | | | -.530 | | | | | |  |
|  |  |  | *A. macularius* | | | | -.449^*^ | | | .027 | | | | **.000** | | | | -.520 | | | | -.377 | | | | | |  |
| *C. semipalmatus* | | | *C. collaris* | | | | .198^*^ | | | .032 | | | | **.000** | | | | .115 | | | | .282 | | | | | |  |
|  |  |  | *C. pusilla* | | | | -.413^*^ | | | .035 | | | | **.000** | | | | -.505 | | | | -.321 | | | | | |  |
|  |  |  | *A. macularius* | | | | -.250^*^ | | | .032 | | | | **.000** | | | | -.335 | | | | -.166 | | | | | |  |
| *C. pusilla* | | | *C. collaris* | | | | .611^*^ | | | .031 | | | | **.000** | | | | .530 | | | | .692 | | | | | |  |
|  |  |  | *C. semipalmatus* | | | | .413^*^ | | | .035 | | | | **.000** | | | | .321 | | | | .505 | | | | | |  |
|  |  |  | *A. macularius* | | | | .163^*^ | | | .031 | | | | **.000** | | | | .081 | | | | .245 | | | | | |  |
| *A. macularius* | | | *C. collaris* | | | | .449^*^ | | | .027 | | | | **.000** | | | | .377 | | | | .520 | | | | | |  |
|  |  |  | *C. semipalmatus* | | | | .250^*^ | | | .032 | | | | **.000** | | | | .166 | | | | .335 | | | | | |  |
|  |  |  | *C. pusilla* | | | | -.163^*^ | | | .031 | | | | **.000** | | | | -.245 | | | | -.081 | | | | | |  |
| Based on estimated marginal means | | | | | | | | | | | | | | | | | | | | | | | | | | | |  |
| *. The average difference is significant at the .05 level. | | | | | | | | | | | | | | | | | | | | | | | | | | | |  |
| a. R squared =.581 (adjusted R squared =.578)  b. Fit for multiple comparisons: Sidak. | | | | | | | | | | | | | | | | | | | | | | | | | | | |  |
