# Supplementary material for: Shorebirds’ Longer Migratory Distances Are Associated With Larger ADCYAP1 Microsatellites and Greater Morphological Complexity of Hippocampal Astrocytes
Source: Front Psychol. 2022 Feb 4;12:784372. doi: 10.3389/fpsyg.2021.784372 (PMC8855117; doi:10.3389/fpsyg.2021.784372)
Supplement: Supplementary file 9 [file Table_9.DOCX]

**S9 Table**. Dispersion Homogeneity Test (PERMIDISP) of astrocytes and paired tests for the interaction of “species / types” factors using Euclidean distances and also the transformation into Log (x + 1) and data normalization.

| DEVIATIONS FROM CENTROID |  |  |  |
| --- | --- | --- | --- |
| F: 5.2778 df1: 7 df2: 1069 |  |  |  |
| P(perm): 0.0001 |  |  |  |
| PAIRWISE COMPARISONS |  |  |  |
| Groups | t | P(perm) |  |
| *(Charadrius collaris_Type1 x Charadrius collaris_Type2)* | 0.15439 | 0.876 |  |
| *(Charadrius collaris_Type1 x Charadrius semipalmatus_Type2)* | 2.1484 | 0.0343 |  |
| *(Charadrius collaris_Type1 x Charadrius semipalmatus_Type1)* | 0.51338 | 0.6254 |  |
| *(Charadrius collaris_Type1 X Calidris pusilla_Type1)* | 1.2902 | 0.2132 |  |
| *(Charadrius collaris_Type1 X Calidris pusilla_Type2)* | 4.4664 | 0.0001 |  |
| *(Charadrius collaris_Type1 X Actitis macularius_Type1)* | 1.3443 | 0.1938 |  |
| *(Charadrius collaris_Type1 X Actitis macularius_Type2)* | 0.28273 | 0.7793 |  |
| *(Charadrius collaris_Type2 X Charadrius semipalmatus_Type2)* | 2.45 | 0.0181 |  |
| *(Charadrius collaris_Type2 X Charadrius semipalmatus_Type1)* | 0.63022 | 0.558 |  |
| *(Charadrius collaris_Type2 X Calidris pusilla_Type1)* | 1.4335 | 0.1618 |  |
| *(Charadrius collaris_Type2 X Calidris pusilla_Type2)* | 4.834 | 0.0001 |  |
| *(Charadrius collaris_Type2 X Actitis macularius_Type1)* | 1.5307 | 0.1355 |  |
| *(Charadrius collaris_Type2 X Actitis macularius_Type2)* | 0.47783 | 0.6474 |  |
| *(Charadrius semipalmatus_Type2 X Charadrius semipalmatus_Type1)* | 0.83362 | 0.4236 |  |
| *(Charadrius semipalmatus_Type2 X Calidris pusilla_Type1)* | 0.32123 | 0.7544 |  |
| *(Charadrius semipalmatus_Type2 X Calidris pusilla_Type2)* | 3.555 | 0.0005 |  |
| *(Charadrius semipalmatus_Type2 X Actitis macularius_Type1)* | 0.1819 | 0.8616 |  |
| *(Charadrius semipalmatus_Type2 X Actitis macularius_Type2)* | 2.1878 | 0.0308 |  |
| *(Charadrius semipalmatus_Type1 X Calidris pusilla_Type1)* | 0.61775 | 0.5829 |  |
| *(Charadrius semipalmatus_Type1 X Calidris pusilla_Type2)* | 2.5661 | 0.0112 |  |
| *(Charadrius semipalmatus_Type1 X Actitis macularius_Type1)* | 0.51465 | 0.6427 |  |
| *(Charadrius semipalmatus_Type1 X Actitis macularius_Type2)* | 0.41492 | 0.6942 |  |
| *(Calidris pusilla_Type1 X Calidris pusilla_Type2)* | 1.4423 | 0.1676 |  |
| *(Calidris pusilla_Type1 X Actitis macularius_Type1)* | 0.31536 | 0.7733 |  |
| *(Calidris pusilla_Type1 X Actitis macularius_Type2)* | 1.3636 | 0.1944 |  |
| *(Calidris pusilla_Type2 X Actitis macularius_Type1)* | 2.5773 | 0.013 |  |
| *(Calidris pusilla_Type2 X Actitis macularius_Type2)* | 5.0151 | 0.0001 |  |
| *(Actitis macularius_Type1 X Actitis macularius_Type2)* | 1.3512 | 0.1924 |  |
|  |  |  |  |
| MEANS AND STANDARD ERRORS |  |  |  |
| Group | Size | Average | SE |
| *Charadrius collaris_Type1* | 120 | 3.2808 | 0.12271 |
| *Charadrius collaris_Type2* | 140 | 3.3067 | 0.11407 |
| *Charadrius semipalmatus_Type2* | 261 | 3.0116 | 6.36E-02 |
| *Charadrius semipalmatus_Type1* | 41 | 3.1591 | 0.1886 |
| *Calidris pusilla_Type1* | 49 | 2.9521 | 0.26255 |
| *Calidris pusilla_Type2* | 202 | 2.6524 | 8.03E-02 |
| *Actitis macularius_Type1* | 96 | 3.0358 | 0.13422 |
| *Actitis macularius_Type2* | 168 | 3.24 | 8.48E-02 |

The values of F and p were obtained using 9999 permutations with distances between centroides for 1077 samples for 8 groups. The p values in red represent statistically significant differences in dispersions.
