# Supplementary material for: Shorebirds’ Longer Migratory Distances Are Associated With Larger ADCYAP1 Microsatellites and Greater Morphological Complexity of Hippocampal Astrocytes
Source: Front Psychol. 2022 Feb 4;12:784372. doi: 10.3389/fpsyg.2021.784372 (PMC8855117; doi:10.3389/fpsyg.2021.784372)
Supplement: Supplementary file 10 [file Table_10.DOCX]

**S10 Table:** FH Astrocyte Dispersion Homogeneity Test (PERMIDISP) and paired tests for the “species” and “types” factors using Euclidean distances and also the transformation into Log (x + 1) and data normalization.

| DEVIATIONS FROM CENTROID |  |  |  |
| --- | --- | --- | --- |
| F: 17.864 df1: 3 df2: 1073 |  |  |  |
| P(perm): **0.0001** |  |  |  |
|  |  |  |  |
| PAIRWISE COMPARISONS |  |  |  |
| Groups | t | P(perm) |  |
| *(Charadrius collaris x Charadrius semipalmatus)* | 4.4893 | **0.0002** |  |
| *(Charadrius collaris x Calidris pusilla)* | 5.7143 | **0.0001** |  |
| *(Charadrius collaris x Actitis macularius)* | 0.71182 | 0.4774 |  |
| *(Charadrius semipalmatus x Calidris pusilla)* | 2.4406 | **0.0158** |  |
| *(Charadrius semipalmatus x Actitis macularius)* | 3.9761 | **0.0003** |  |
| *(Calidris pusilla x Actitis macularius)* | 5.3787 | **0.0001** |  |
|  |  |  |  |
| MEANS AND STANDARD ERRORS |  |  |  |
| Group | Size | Average | SE |
| *Charadrius collaris* | 260 | 3.6862 | 9.26E-02 |
| *Charadrius semipalmatus* | 302 | 3.19E+00 | 6.30E-02 |
| *Calidris pusilla* | 251 | 2.9216 | 9.67E-02 |
| *Actitis macularius* | 264 | 3.5986 | 8.13E-02 |
|  |  |  |  |
|  |  |  |  |
| DEVIATIONS FROM CENTROID |  |  |  |
| F: 0.94848 df1: 1 df2: 1075 |  |  |  |
| P(perm): 0.3381 |  |  |  |
| PAIRWISE COMPARISONS |  |  |  |
| Groups | t | P(perm) |  |
| (Type1 X Type2) | 0.9739 | 0.3386 |  |
| MEANS AND STANDARD ERRORS |  |  |  |
| Group | Size | Average | SE |
| Type1 | 306 | 3.748 | 8.51E-02 |
| Type2 | 771 | 3.663 | 4.35E-02 |

Os valores de *F* e de *p* foram obtidos usando 9999 permutações com distâncias entre centroides apara 1077 amostras para 4 grupos nas espécies e 2 grupos nos tipos. Os valores de p em vermelho representam diferenças estatisticamente significativas nas dispersões.
