# Supplementary material for: Shorebirds’ Longer Migratory Distances Are Associated With Larger ADCYAP1 Microsatellites and Greater Morphological Complexity of Hippocampal Astrocytes
Source: Front Psychol. 2022 Feb 4;12:784372. doi: 10.3389/fpsyg.2021.784372 (PMC8855117; doi:10.3389/fpsyg.2021.784372)
Supplement: Supplementary file 11 [file Table_11.DOCX]

**S11 Table:** Analysis of Variance of Multivariate Permutation of Astrocytes Morphometric Features through the factors "species" and "types" of astrocytes.

|  |  |  |  |  |  | Unique |  |
| --- | --- | --- | --- | --- | --- | --- | --- |
| Source | df | SS | MS | Pseudo-F | P(perm) | perms | P(MC) |
| Species | 3 | 3503.1 | 1167.7 | 106.82 | 0.0001 | 9926 | 0.0001 |
| Type | 1 | 2040.9 | 2040.9 | 186.69 | 0.0001 | 9964 | 0.0001 |
| Species x Type | 3 | 1.80E+02 | 59.903 | 5.4797 | 0.0001 | 9913 | 0.0001 |
| Res | 1069 | 11686.000 | 1.09E+01 |  |  |  |  |
| Total | 1076 | 18292.000 |  |  |  |  |  |
|  |  |  |  |  |  |  |  |
| Details of the expected mean squares (EMS) for the model | | |  |  |  |  |  |
| Source | EMS |  |  |  |  |  |  |
| Especie | 1*V(Res) + 196.18*S(Es) | |  |  |  |  |  |
| Type | 1*V(Res) + 374.6*S(Ti) | |  |  |  |  |  |
| EspeciexType | 1*V(Res) + 98.092*S(EsxTi) | |  |  |  |  |  |
| Res | 1*V(Res) |  |  |  |  |  |  |
|  |  |  |  |  |  |  |  |
| Construction of Pseudo-F ratio(s) from mean squares | | |  |  |  |  |  |
| Source | Numerator | Denominator | Num.df | Den.df |  |  |  |
| Especie | 1*Es | 1*Res | 3 | 1069 |  |  |  |
| Type | 1*Ti | 1*Res | 1 | 1069 |  |  |  |
| EspeciexType | 1*EsxTi | 1*Res | 3 | 1069 |  |  |  |
|  |  |  |  |  |  |  |  |
| Estimates of components of variation | |  |  |  |  |  |  |
| Source | Estimate | Sq.root |  |  |  |  |  |
| S(Es) | 5.8962 | 2.4282 |  |  |  |  |  |
| S(Ti) | 5.419 | 2.3279 |  |  |  |  |  |
| S(EsxTi) | 0.49923 | 0.70656 |  |  |  |  |  |
| V(Res) | 10.932 | 3.3063 |  |  |  |  |  |

The pseudo - F statistics were calculated for each term using direct analogues for univariate mean square expectations (EMS); p values were obtained using 9999 permutations under a reduced model. The sum of squares was considered partial (type III) and each term is identified as contributing a fixed component to the general model; "Var" provides the estimated sizes of the components of the variation, based on multivariate analogs for the classic ANOVA unbiased estimators; "SD" gives the square root of these values. The data were transformed into Log (x + 1) and normalized.
