# Supplementary material for: Shorebirds’ Longer Migratory Distances Are Associated With Larger ADCYAP1 Microsatellites and Greater Morphological Complexity of Hippocampal Astrocytes
Source: Front Psychol. 2022 Feb 4;12:784372. doi: 10.3389/fpsyg.2021.784372 (PMC8855117; doi:10.3389/fpsyg.2021.784372)
Supplement: Supplementary file 12 [file Table_12.DOCX]

**S12 Table:** PERMANOVA of Astrocytes for paired tests through the factors "species" and "types" of astrocytes found in FH for the 17 morphometric characteristics analyzed.

| PAIR-WISE TESTS |  |  |  |  |  |
| --- | --- | --- | --- | --- | --- |
|  |  |  |  |  |  |
| Term 'EsxTi' for pairs of levels of factor 'Especie' |  |  |  |  |  |
| Within level '1' of factor 'Tipo' |  |  |  |  |  |
| Groups | t | P(perm) | perms | P(MC) |  |
| *Charadrius collaris, Charadrius semipalmatus* | 2.6457 | 0.0003 | 9.93E+03 | 0.0002 |  |
| *Charadrius collaris, Calidris pusilla* | 8.3178 | 0.0001 | 9931 | 0.0001 |  |
| *Charadrius collaris, Actitis macularius* | 7.7342 | 0.0001 | 9945 | 0.0001 |  |
| *Charadrius semipalmatus, Calidris pusilla* | 6.0436 | 0.0001 | 9948 | 0.0001 |  |
| *Charadrius semipalmatus, Actitis macularius* | 4.2455 | 0.0001 | 9.94E+03 | 0.0001 |  |
| *Calidris pusilla, Actitis macularius* | 4.5504 | 0.0001 | 9.95E+03 | 0.0001 |  |
|  |  |  |  |  |  |
| Denominators |  |  |  |  |  |
| Groups | Denominator | Den.df |  |  |  |
| *(Charadrius collaris x Charadrius semipalmatus)* | 1*Res | 159 |  |  |  |
| *(Charadrius collaris x Calidris pusilla)* | 1*Res | 167 |  |  |  |
| *(Charadrius collaris x Actitis macularius)* | 1*Res | 214 |  |  |  |
| *(Charadrius semipalmatus x Calidris pusilla)* | 1*Res | 88 |  |  |  |
| *(Charadrius semipalmatus x Actitis macularius)* | 1*Res | 135 |  |  |  |
| *(Calidris pusilla x Actitis macularius)* | 1*Res | 143 |  |  |  |
|  |  |  |  |  |  |
| Average Distance between/within groups |  |  |  |  |  |
|  | Charadrius collaris | Charadrius semipalmatus | Calidris pusilla | Actitis macularius |  |
| *Charadrius collaris* | 4.6824 |  |  |  |  |
| *Charadrius semipalmatus* | 4.9029 | 4.5288 |  |  |  |
| *Calidris pusilla* | 6.6623 | 6.2606 | 4.2509 |  |  |
| *Actitis macularius* | 5.7518 | 5.1262 | 5.1163 | 4.3745 |  |
|  |  |  |  |  |  |
| Within level '2' of factor 'Tipo' |  |  |  |  |  |
|  |  |  | Unique |  |  |
| Groups | t | P(perm) | perms | P(MC) |  |
| *(Charadrius collaris x Charadrius semipalmatus)* | 9.5098 | 0.0001 | 9953 | 0.0001 |  |
| *(Charadrius collaris x Calidris pusilla)* | 14.478 | 0.0001 | 9956 | 0.0001 |  |
| *(Charadrius collaris x Actitis macularius)* | 9.0278 | 0.0001 | 9947 | 0.0001 |  |
| *(Charadrius semipalmatus x Calidris pusilla)* | 13.914 | 0.0001 | 9955 | 0.0001 |  |
| *(Charadrius semipalmatus x Actitis macularius)* | 4.9716 | 0.0001 | 9936 | 0.0001 |  |
| *(Calidris pusilla x Actitis macularius)* | 7.6944 | 0.0001 | 9942 | 0.0001 |  |
|  |  |  |  |  |  |
| Denominators |  |  |  |  |  |
| Groups | Denominator | Den.df |  |  |  |
| *(Charadrius collaris x Charadrius semipalmatus)* | 1*Res | 399 |  |  |  |
| *(Charadrius collaris x Calidris pusilla)* | 1*Res | 340 |  |  |  |
| *(Charadrius collaris x Actitis macularius)* | 1*Res | 306 |  |  |  |
| *(Charadrius semipalmatus x Calidris pusilla)* | 1*Res | 461 |  |  |  |
| *(Charadrius semipalmatus x Actitis macularius)* | 1*Res | 427 |  |  |  |
| *(Calidris pusilla x Actitis macularius)* | 1*Res | 368 |  |  |  |
|  |  |  |  |  |  |
| Average Distance between/within groups |  |  |  |  |  |
|  | Charadrius collaris | Charadrius semipalmatus | Calidris pusilla | Actitis macularius | |
| *Charadrius collaris* | 4.6931 |  |  |  |  |
| *Charadrius semipalmatus* | 5.4811 | 4.2583 |  |  |  |
| *Calidris pusilla* | 6.6025 | 5.7197 | 3.7916 |  |  |
| *Actitis macularius* | 5.7909 | 4.7313 | 4.9195 | 4.5824 |  |
|  |  |  |  |  |  |
| PAIR-WISE TESTS |  |  |  |  |  |
| Term 'Es' |  |  |  |  |  |
|  |  |  | Unique |  |  |
| Groups | t | P(perm) | perms | P(MC) |  |
| *Charadrius collaris, Charadrius semipalmatus* | 6.7191 | 0.0001 | 9.95E+03 | 0.0001 |  |
| *Charadrius collaris, Calidris pusilla* | 15.005 | 0.0001 | 9.94E+03 | 0.0001 |  |
| *Charadrius collaris, Actitis macularius* | 11.63 | 0.0001 | 9951 | 0.0001 |  |
| *Charadrius semipalmatus, Calidris pusilla* | 11.285 | 0.0001 | 9928 | 0.0001 |  |
| *Charadrius semipalmatus, Actitis macularius* | 5.5313 | 0.0001 | 9948 | 0.0001 |  |
| *Calidris pusilla, Actitis macularius* | 7.7212 | 0.0001 | 9.95E+03 | 0.0001 |  |
|  |  |  |  |  |  |
| Denominators |  |  |  |  |  |
| Groups | Denominator | Den.df |  |  |  |
| *Charadrius collaris, Charadrius semipalmatus* | 1*Res | 558 |  |  |  |
| *Charadrius collaris, Calidris pusilla* | 1*Res | 507 |  |  |  |
| *Charadrius collaris, Actitis macularius* | 1*Res | 520 |  |  |  |
| *Charadrius semipalmatus, Calidris pusilla* | 1*Res | 549 |  |  |  |
| *Charadrius semipalmatus, Actitis macularius* | 1*Res | 562 |  |  |  |
| *Calidris pusilla, Actitis macularius* | 1*Res | 511 |  |  |  |
|  |  |  |  |  |  |
| Average Distance between/within groups |  |  |  |  |  |
|  | Charadrius collaris | Charadrius semipalmatus | Calidris pusilla | Actitis macularius | |
| *Charadrius collaris* | 5.2416 |  |  |  |  |
| *Charadrius semipalmatus* | 5.3487 | 4.5204 |  |  |  |
| *Calidris pusilla* | 6.4433 | 6.024 | 4.2182 |  |  |
| *Actitis macularius* | 6.006 | 5.2983 | 5.3269 | 5.0977 |  |
